# Enhancing Clinical Detection Accuracy of Large Structured Viral RNA via DNAzyme Cleavage and Antisense‐Assisted Rolling Circle Amplification

**DOI:** 10.1002/anie.202507973

**Published:** 2025-06-26

**Authors:** Amal Mathai, Jimmy Gu, Connor Nurmi, John D. Brennan, Yingfu Li

**Affiliations:** ^1^ Department of Biochemistry and Biomedical Sciences McMaster University 1280 Main Street West Hamilton ON L8S 4K1 Canada; ^2^ Biointerfaces Institute McMaster University 1280 Main Street West Hamilton ON L8S 4M1 Canada

**Keywords:** Antisense oligonucleotides, Biosensors, DNAzymes, Rolling circle amplification, Structural elements

## Abstract

Sensitive detection of viral RNA is critical for accurate diagnostic testing, particularly during outbreaks of emerging infectious diseases. Rolling circle amplification (RCA) is a powerful isothermal amplification strategy that can be directly primed by RNA, eliminating the need for reverse transcription. Previous approaches have used 10–23 DNAzymes to cleave viral RNA, generating 3′‐ends for hybridization to circular DNA templates (CDTs). However, the resulting RNA fragments often retained secondary or tertiary structures that hindered CDT binding and limited RCA efficiency. To address this challenge, we developed antisense oligonucleotide‐assisted RCA (ASO‐RCA), a general strategy that uses short upstream antisense oligonucleotides (ASOs) to remodel RNA structure and expose the CDT‐binding site. Using five DNAzyme‐CDT systems targeting distinct regions of the SARS‐CoV‐2 genome, we show that ASO inclusion improves CDT hybridization and enhances RCA output—by up to 70‐fold. This enhancement was observed using both linear and quasi‐exponential RCA formats and remained effective in 50% pooled saliva. When applied to clinical saliva samples, ASO‐assisted RCA markedly improved diagnostic performance, achieving 100% sensitivity and up to 97.5%–100% accuracy across multiple systems. These findings establish ASO‐DNAzyme‐RCA as a simple, robust, and clinically relevant platform for improving nucleic acid detection in structured RNA targets.

## Graphical abstract

We show that antisense oligonucleotides can be used to restructure secondary structural elements around the circular DNA template binding sites of the genomic RNA for subsequent rolling circle amplification. This strategy was employed to identify otherwise COVID “false negative” saliva samples by accurately detecting the presence of SARS‐CoV‐2 genomic RNA in patient saliva samples, resulting in improved clinical sensitivity and specificity.

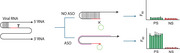

## Introduction

Highly sensitive biosensing technologies are essential for accurate and timely disease diagnosis, particularly when biomarkers are present at low concentrations.^[^ ^1^, ^2^, ^3^, ^4^ ^]^ To improve detection sensitivity, amplification strategies are often incorporated into nucleic‐acid‐based biosensors. Polymerase chain reaction (PCR) remains the gold standard for nucleic acid detection due to its high sensitivity and specificity.^[^ ^5^, ^6^, ^7^, ^8^ ^]^ However, PCR requires thermal cycling, sophisticated instrumentation, and trained personnel, making it impractical for point‐of‐care (POC) applications. In response, various isothermal amplification (ITA) methods, such as loop‐mediated isothermal amplification (LAMP),^[^ ^9^, ^10^, ^11^, ^12^ ^]^ strand displacement amplification (SDA),^[^ ^13^, ^14^, ^15^, ^16^ ^]^ and recombinase polymerase amplification (RPA),^[^ ^17^, ^18^, ^19^ ^]^ have been developed as alternatives.^[^ ^20^ ^]^ Although these methods are promising for POC use, many still rely on multiple enzymes, complex primer designs, and elevated temperatures (30–65 °C), limiting their accessibility and robustness in resource‐limited settings.^[^ ^21^, ^22^ ^]^

Rolling circle amplification (RCA) is a particularly attractive ITA approach because it operates at room temperature, requires only a single enzyme (phi29 DNA polymerase), and can be directly initiated using RNA or DNA primers, thus eliminating the need for reverse transcription for RNA detection.^[^ ^23^, ^24^, ^25^, ^26^, ^27^, ^28^, ^29^, ^30^, ^31^, ^32^, ^33^ ^]^ RCA is initiated when a primer hybridizes to a circular DNA template (CDT), enabling continuous synthesis of long, tandem‐repeat single‐stranded DNA that can be easily detected by fluorescence or colorimetric methods. This makes RCA a simple, cost‐effective, and powerful platform for molecular diagnostics.^[^ ^23^ ^]^

Although RCA typically relies on short synthetic DNA primers, our group previously developed a strategy in which RNA‐cleaving DNAzymes (RCDs) generated defined RNA fragments from large, structured viral genomic RNAs (gRNAs), enabling these fragments to serve as primers for RCA initiation.^[^ ^34^ ^]^ By cleaving gRNA at specific sites, DNAzymes create 3′ termini that can hybridize with CDTs to trigger amplification. However, despite screening 230 DNAzyme candidates across the SARS‐CoV‐2 genome and identifying 34 with strong cleavage activity, only 8 of these systems successfully initiated RCA—and just one generated sufficient signal for clinical application.^[^ ^34^ ^]^ This low success rate pointed to a critical limitation: the RNA fragments, though correctly cleaved, often retained stable secondary or tertiary structures that occluded the CDT‐binding region, preventing effective primer–template hybridization and thereby limiting RCA performance.

To overcome this barrier, we developed a targeted solution—antisense oligonucleotide‐assisted RCA (ASO‐RCA)—which enhances primer–template hybridization by locally restructuring the RNA near the CDT‐binding site. Antisense oligonucleotides (ASOs) are short synthetic sequences that bind complementary regions of RNA and remodel local structures.^[^ ^35^ ^]^ In our prior work, we showed that ASOs could enhance DNAzyme cleavage of long structured RNAs (lsRNAs) by up to 2000‐fold by disrupting stable secondary structures and improving access to the DNAzyme recognition site.^[^ ^36^ ^]^ Although ASOs have also been used to modulate ribozyme activity,^[^ ^37^ ^]^ gene expression,^[^ ^38^, ^39^, ^40^, ^41^, ^42^, ^43^, ^44^, ^45^, ^46^, ^47^, ^48^, ^49^, ^50^ ^]^ and biosensor performance,^[^ ^51^, ^52^, ^53^, ^54^, ^55^, ^56^ ^]^ their use to facilitate primer binding and nucleic acid amplification from structured RNA targets has not been reported. This study introduces, for the first time, a generalizable strategy that applies ASOs to unlock rolling circle amplification from DNAzyme‐cleaved, structured viral RNAs, addressing a key barrier in RNA‐primed isothermal amplification.

Here, we demonstrate that rationally designed ASOs can effectively restructure DNAzyme‐cleaved RNA fragments to expose the CDT‐binding site and enable efficient RCA initiation. Because CDT hybridization occurs at the 3′ end of the cleaved RNA, only upstream ASOs are required—a unique constraint that distinguishes this strategy from our previous ASO application targeting DNAzyme cleavage efficiency. We applied ASO‐RCA to five DNAzyme‐CDT systems targeting different regions of the SARS‐CoV‐2 genome, each previously limited by poor RCA performance despite strong DNAzyme cleavage. In all cases, ASO incorporation led to substantial improvements in amplification efficiency. Moreover, ASO‐RCA enabled sensitive and specific detection of SARS‐CoV‐2 RNA in unprocessed patient saliva samples, demonstrating robust performance even in complex clinical matrices. Together, these results establish ASO‐RCA as a broadly applicable and clinically relevant strategy for enhancing isothermal amplification from structured RNA, with strong potential to advance molecular diagnostics.

## Results and Discussion

### Selection of RCA Coupling Systems

To evaluate the general applicability of ASOs in enhancing RCA from structured RNA targets, we selected five representative DNAzyme‐RCA coupling systems from our previously reported panel of 34 designs.^[^ ^34^ ^]^ These systems, denoted as Systems 1–5, consist of: ls584 with dZ_12618a and CDT1 (System 1), ls1798 with dZ_4148a and CDT2 (System 2), ls1208 with dZ_13726a and CDT3 (System 3), ls1805 with dZ_17522a and CDT4 (System 4), and ls831 with dZ_25806a and CDT5 (System 5). Full sequences for the RNA transcripts, DNAzymes, and CDTs are provided in Table S1.

Each system is named using a standardized convention: the prefix “ls” refers to large, structured RNA (lsRNA) derived from the SARS‐CoV‐2 genome, with the number indicating transcript length in nucleotides. DNAzymes are labeled “dZ” followed by the cleavage site position relative to the reference genome. Each CDT is designed to hybridize with the final 20 nucleotides at the 3′ end of the 5′ cleavage product.

**Figure 1:**
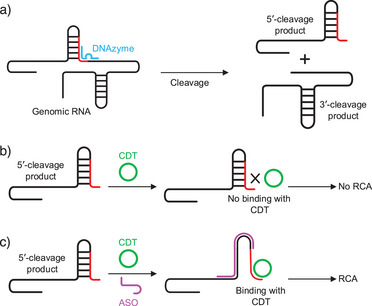
Conceptual schematic of antisense oligonucleotide‐assisted rolling circle amplification (ASO‐RCA) for detecting structured genomic RNA. a) A 10–23 DNAzyme cleaves structured viral genomic RNA at a defined site, generating 5′ and 3′ cleavage products. b) In the absence of an antisense oligonucleotide (ASO), the 5′ cleavage product retains secondary structures that occlude the CDT‐binding region (shown in red), preventing effective hybridization with the circular DNA template (CDT) and thereby inhibiting RCA initiation. c) When a rationally designed ASO (magenta) is added, it binds upstream of the CDT‐binding site, disrupting inhibitory RNA structures and exposing the 3′ end for CDT hybridization. This structural remodeling enables efficient primer–template pairing and successful RCA initiation by phi29 DNA polymerase.

The RNA sequences correspond to different regions of the SARS‐CoV‐2 genome, including fragments from NSP8, NSP3, NSP12, NSP13, and ORF3a, and were synthesized from PCR‐derived templates.^[^ ^34^ ^]^ The selected systems span a range of RCA coupling efficiencies. Systems 1–3 exhibited high coupling, producing fluorescence enhancements of 5000 to 15 000 RFU following DNAzyme cleavage, whereas Systems 4 and 5 showed relatively low RCA signals (<5000 RFU), as detailed in Table S2. Notably, all five systems demonstrated robust DNAzyme cleavage activity, as reflected by previously reported *k*_obs_ values,^[^ ^34^ ^]^ ensuring that the main performance bottleneck lies in the accessibility of the CDT‐binding region on the cleaved RNA. This distinction provided a suitable testbed to investigate whether ASOs could resolve RNA structural barriers and enhance RCA efficiency, as illustrated in Figure 1.

### Designing ASOs to Improve CDT Accessibility to Cleaved lsRNA

To evaluate whether ASOs can enhance RCA efficiency by improving CDT accessibility, we first analyzed the secondary structures of lsRNAs used in the five selected DNAzyme–RCA coupling systems. The minimum free energy structures and base‐pairing probabilities were predicted using the Mathews Lab RNAstructure webserver. A detailed analysis of System 1 (ls584/dZ_12618a/CDT1) is presented here as a representative example, whereas analogous structural insights for Systems 2–5 are included in the Supporting Information.

**Figure 2:**
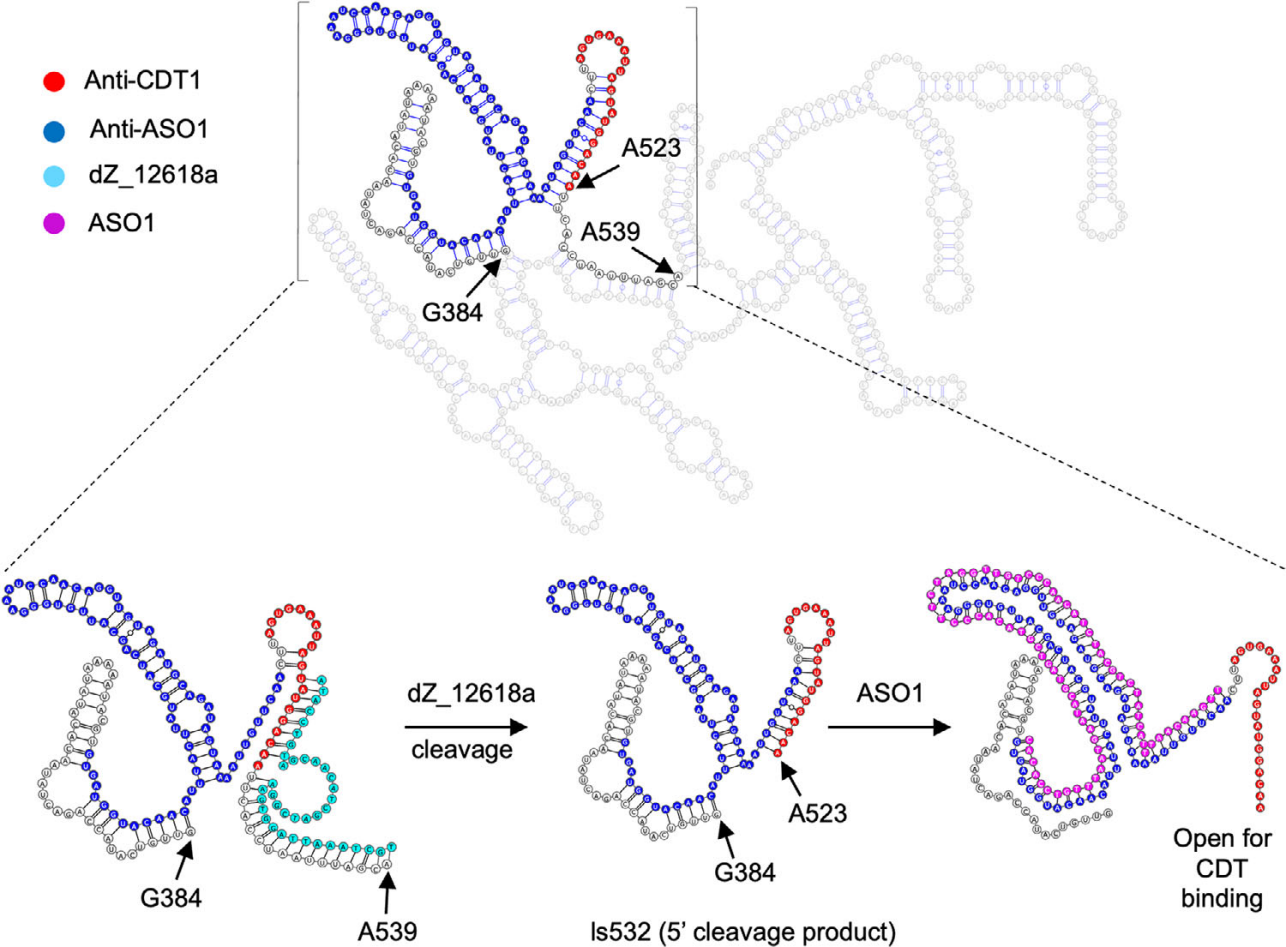
Secondary structure analysis of the ls584 RNA transcript (System 1) and the mechanism by which ASO1 enhances accessibility to the CDT1‐binding region. The full secondary structure of ls584, predicted using the Mathews Lab RNAstructure software, is shown at the top, with the CDT1‐binding region (Anti‐CDT1) in red and the ASO1‐binding region (Anti‐ASO1) in blue. The bottom‐left structure shows the DNAzyme dZ_12618a (shown in cyan) bound to ls584, cleaving the RNA between A523 and U524. The resulting 5′‐cleavage product (bottom center) retains significant secondary structure, with the Anti‐CDT1 region (red) still partially sequestered. Upon addition of ASO1 (magenta, bottom‐right), the upstream RNA structure is reorganized, freeing the Anti‐CDT1 region and enabling effective hybridization with the circular DNA template (CDT) for RCA initiation.

Figure 2 illustrates the structural context of System 1. The full‐length ls584 transcript (584 nt) contains a red region targeted by CDT1 (20 nt) and a blue region (Anti‐ASO1) that serves as the binding site for ASO1 (80 nt). DNAzyme binding and cleavage occur between nucleotides A523 and U524, generating a 5′‐cleavage product denoted as ls532. This cleavage product contains the red‐marked Anti‐CDT1 region, designed to hybridize with CDT1 to initiate RCA. However, secondary structure predictions reveal that this region remains partially sequestered due to strong base pairing with a portion of the Anti‐ASO1 region, forming a stable stem‐loop that inhibits effective CDT binding. To address this structural occlusion, we designed ASO1 to target the entire Anti‐ASO1 region immediately upstream of the Anti‐CDT1 region. As shown in the bottom‐right panel of Figure 2, ASO1 binding (magenta) disrupts local intramolecular base pairing, liberating the Anti‐CDT1 region and allowing it to adopt an open conformation suitable for CDT hybridization and RCA initiation. Notably, to avoid steric overlap and competition between CDT1 and ASO1, three nucleotides adjacent to the Anti‐CDT1 sequence were intentionally excluded from the ASO1 design. This upstream‐only ASO placement strategy was consistently applied across all five systems, with similar effects observed in Systems 2–5 (Figures S1–S4). These results demonstrate a general design principle: strategic ASO binding upstream of the CDT target site can resolve inhibitory RNA structures, thereby enabling efficient primer–template hybridization and increasing RCA performance. Use of multiple ASOs, as reported in our previous study on improving DNAzyme accessibility,^[^ ^36^ ^]^ was avoided as it was expected that a single ASO binding upstream of the CDT binding site should release the 3′ end of the cleavage product to allow CDT hybridization and initiate RCA. In addition, incorporating additional ASOs may increase the likelihood of nonspecific interactions with the CDT, potentially leading to higher background signals.

### ASO Impact on Binding Between CDT and RNA

To validate that ASOs improve CDT binding by disrupting inhibitory RNA structures, we performed native polyacrylamide gel electrophoresis (nPAGE) assays using a truncated 63 nt RNA construct containing both the CDT‐ and ASO‐binding regions from System 1. The use of a shortened RNA fragment was necessary because gel mobility shifts between full‐length RNA (e.g., 584–664 nt) are difficult to resolve clearly on nPAGE, whereas the mobility shifts between 63 and 143 nt are more easily detectable. Importantly, despite its shorter length, the 63 nt RNA retains native secondary structure that can be targeted by the full‐length 80 nt ASO1.

**Figure 3:**
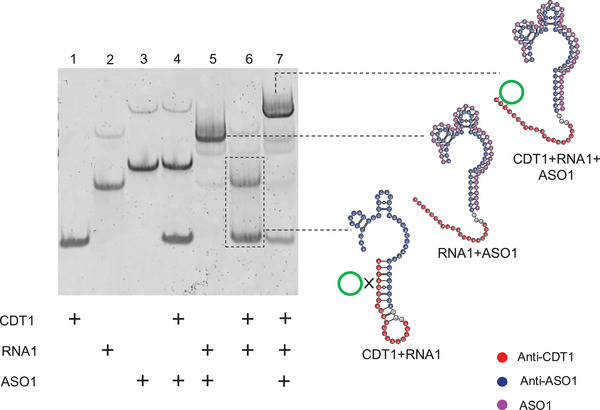
Native gel shift assay demonstrating that ASO1 enhanced hybridization between CDT1 and structured RNA1. In lane 6, CDT1 was incubated with 63 nt RNA1 alone, resulting in minimal complex formation, as indicated by the predominance of unshifted CDT1. In contrast, lane 7 showed that the addition of ASO1 promoted structural reorganization of RNA1, enabling efficient binding to CDT1 and resulting in a prominent shifted band. Corresponding secondary structure models (right) illustrate how ASO1 binding (magenta) exposed the Anti‐CDT1 region (red) by disrupting competing intramolecular structures formed by the Anti‐ASO1 region (blue), thus facilitating CDT hybridization.

As shown in Figure 3, lanes 1–3 included controls for CDT1, RNA1, and ASO1 alone, respectively. Lane 4 confirmed that ASO1 did not bind CDT1 as no mobility shift was observed when the two were incubated together, thereby ruling out nonspecific interactions that could result in background amplification or unintended primer–template binding. This finding is further supported by results from Figure S5, which shows that across all five systems tested, none of the ASOs exhibited binding to their respective CDTs in native gel shift assays. These data confirm that the ASOs do not act as primers or form stable complexes with the CDT, and their effect on RCA efficiency is solely due to structural reorganization of the RNA substrate.

In lane 5, a clear shift indicated the formation of the RNA1–ASO1 complex. In the absence of ASO1 (lane 6), minimal binding occurred between RNA1 and CDT1, with most of the signal remaining in the unbound RNA and CDT bands—consistent with predicted RNA secondary structures sequestering the CDT‐binding site. In contrast, upon addition of ASO1 (lane 7), a distinct band corresponding to the RNA1–ASO1–CDT1 tripartite complex appeared, accompanied by reduced intensity of the unbound species. This shift confirmed that ASO1 binding promoted structural reorganization of the RNA, exposing the Anti‐CDT1 region for hybridization. An intermediate band corresponding to the RNA1–ASO1 complex was also observed, indicating that some RNA remained bound only to ASO1. Similar behavior was observed across all five systems (Figures S6–S9), demonstrating that rationally designed ASOs consistently resolved structural barriers and facilitated robust CDT binding in both short and long RNA targets.

### ASO Impact on RCA Coupling

To evaluate the effect of ASOs on RCA efficiency, we first optimized the length and concentration of ASO required to effectively disrupt inhibitory secondary structures in lsRNA. RCA reactions were conducted following a 30‐min DNAzyme cleavage step (5 nM lsRNA), during which various ASO lengths (20–80 nt) and concentrations (1–25 nM) were tested. RCA was initiated by adding a relevant CDT, dNTPs, Syto9 dye, and each ASO variant. Importantly, ASOs were added only after the cleavage step to avoid interference with DNAzyme activity. Moreover, each ASO was designed to bind >24 nt upstream of the cleavage site, minimizing any direct influence on cleavage efficiency, as supported by our earlier findings.^[^ ^36^ ^]^

Fluorescence resulting from Syto9 binding to RCA products was recorded over 60 min. The strongest RCA signal, measured at 30 min (*F*_30_), was obtained using an 80 nt ASO at 25 nM (Figure S10). This length fully covers the structured region predicted to inhibit CDT binding, whereas shorter ASOs only partially disrupt these secondary structures. Extending the ASO length beyond 80 nt did not lead to a further increase in RCA signal and instead resulted in a slight decrease. This may be attributed to the increased probability of longer ASOs forming intramolecular secondary structures, which could compromise their ability to effectively bind to the structured regions of the lsRNA and enhance CDT accessibility. A 5:1 ASO/RNA ratio also ensured complete hybridization and maximum RCA efficiency. All subsequent experiments used 80 nt ASOs at 25 nM.

**Figure 4:**
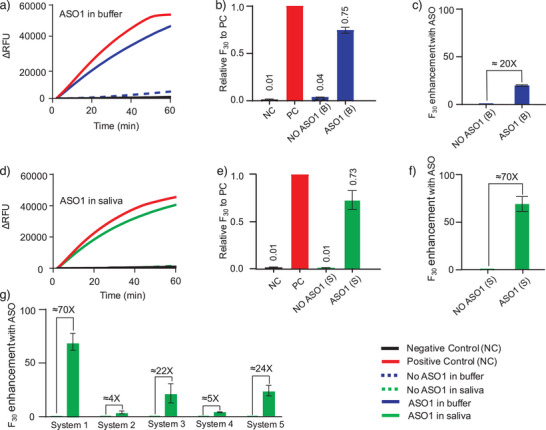
ASO1 enhances the RCA signal following DNAzyme‐mediated cleavage of structured RNA (System 1) in both buffer and 50% pooled negative saliva. a) Real‐time RCA fluorescence curves show that ASO1 substantially increases signal output compared to reactions without ASO1. b) Relative fluorescence units (RFU) at 30 min (*F*_30_) normalized to the positive control (PC) in buffer. c) Fold enhancement in RFU by ASO1, calculated by dividing *F*_30_ with ASO1 by *F*_30_ without ASO1. Equivalent analyses in 50% negative pooled saliva. d) RCA fluorescence curves, e) relative *F*_30_ to PC, and f) fold enhancement with ASO1, showing signal improvements even in complex clinical matrices. g) Summary bar plots showing ASO‐mediated signal enhancement (*F*_30_‐fold change) across five different DNAzyme‐RCA systems in 50% pooled saliva, highlighting the general applicability of ASO‐assisted RCA for structured RNA detection.

Figure 4a shows the performance of System 1 in buffer, where ASO1 dramatically improved RCA output compared to reactions without ASO. The relative *F*_30_ values, normalized to the positive control (PC; unstructured RNA), are shown in Figure 4b. Without ASO1, RCA efficiency was only 4% of the PC, whereas with ASO1 it increased to 75%. This corresponds to a ∼20‐fold enhancement in RCA output (Figure 4c). Similar buffer‐based results for the remaining systems are presented in Figure S11. Across all five systems, ASOs enhanced RCA performance by 5‐ to 41‐fold, depending on the system. Notably, Systems 1, 3, and 5 reached *F*_30_ values >50% of the PC, consistent with substantial improvement in CDT hybridization due to RNA structural remodeling by ASOs.

Figure S12 shows RCA products generated under four reaction conditions across the five tested systems, resolved on a 1.5% agarose gel (lanes 2–5). These products were subsequently digested with the FastDigest BamHI restriction enzyme (lanes 6–9) into monomeric units. Lane 1 contains DNA ladders corresponding to 700, 500, 400, 300, 200, 150, 100, 75, 50, and 25 base pairs, respectively. Distinct bands corresponding to 51‐nt monomeric RCA products were observed in both the positive control and ASO‐containing reactions. In contrast, undigested reactions produced DNA bands near the position of loading wells, indicative of high‐molecular‐weight concatemeric DNA. Notably, the presence of much stronger RCA product bands in the ASO reactions across all systems demonstrates an enhancement in RCA yield compared to reactions performed without ASOs.

To assess ASO function in a biologically relevant context, we repeated the RCA assays in 50% pooled negative saliva. As shown in Figure 4d–f, System 1 again demonstrated significant ASO1‐mediated signal enhancement, with RCA efficiency increasing from near‐zero to 73% of the PC, representing a ∼70‐fold improvement. Similar effects were observed for Systems 2–5 (Figure S13), although Systems 4 and 5 showed somewhat reduced performance compared to buffer, likely due to inhibitory components in saliva or incomplete structural disruption. The comparative RCA enhancement across all five systems in saliva is summarized in Figure 4g. ASO‐mediated improvements ranged from ∼4‐fold in System 2 to ∼70‐fold in System 1, indicating that ASOs provide a robust and generalizable strategy for overcoming structural barriers to RCA, even in complex clinical matrices. It was generally observed that larger RNA transcripts tended to form more complex secondary structures, which may impact ASO and/or CDT binding efficiency. However, further studies are required to determine whether RNA length is a significant factor influencing RCA performance.

The selectivity of RCA detection has been well‐documented in previous studies, demonstrating that the circular DNA template (CDT) exhibits high specificity toward its complementary primer.^[^ ^57^ ^]^ Alterations in the primer sequence typically disrupt hybridization with the CDT, thereby preventing RCA initiation. To experimentally assess cross‐reactivity, unstructured primers previously used as positive controls in the fluorescence‐based RCA assay were added with all five CDTs. For instance, primer 1—complementary only to CDT1—was tested against CDTs 2 through 5. Fluorescence from Syto9 binding to amplified products was monitored over 60 min. As seen in Figure S14A–E, signal was observed exclusively when primers matched their respective CDTs, confirming the high target selectivity of the CDTs and reinforcing the specificity of RCA‐based detection. Fluorescence signals at 60 min (*F*_60_) for all other systems are summarized in Figure S14F.

### Clinical Validation

To evaluate the clinical utility of ASO‐assisted RCA systems for detecting genomic RNA in patient samples, we employed a quasi‐exponential RCA (QE‐RCA) strategy as described previously (see schematic in Figure S15), in which linear RCA is first initiated by a DNAzyme‐cleaved RNA fragment, and a secondary primer (P2) subsequently binds the RCA product to trigger a second round of amplification, thereby enhancing signal output.^[^ ^34^ ^]^ Before analyzing clinical samples, we validated the performance of each of the five systems in 50% pooled negative saliva spiked with the appropriate cleaved lsRNA target. As shown in Figure S16, inclusion of the corresponding ASO led to significant improvements in RCA output, with fold enhancements ranging from ∼2‐fold (System 5) to ∼27‐fold (System 4). Although these fold increases were slightly lower than those observed under linear RCA conditions (Figure S12C), the overall output was higher due to amplification from the QE‐RCA mechanism. This supports the hypothesis that ASOs act specifically at the primer–CDT binding step and do not interfere with downstream amplification steps such as P2 re‐priming.

**Figure 5:**
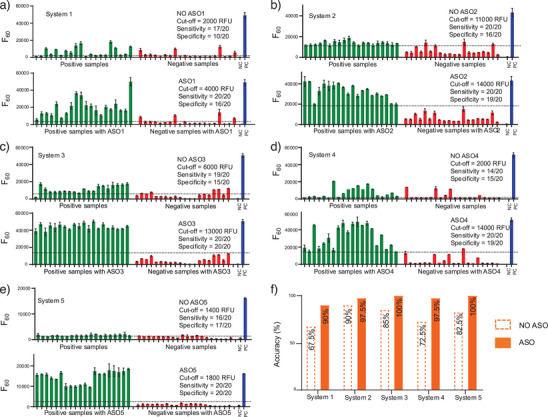
ASOs improve sensitivity, specificity, and overall diagnostic accuracy in clinical SARS‐CoV‐2 detection using five DNAzyme‐RCA systems. a)–e) Bar plots showing *F*_60_ values (fluorescence at 60 min) for 20 positive samples (PS) and 20 negative samples (NS) tested with and without their respective ASOs for Systems 1–5. Each dotted line indicates the assay‐specific fluorescence cut‐off value used to distinguish positive and negative results. Inclusion of ASOs consistently elevated signal in PS and reduced background in NS, leading to improved assay performance. Error bars represent one standard deviation from triplicate measurements. NC, negative control (CDT only, no target RNA); PC, positive control (maximum RCA signal in buffer with ASO). f) Bar graph summarizing diagnostic accuracy (%) for each system with and without ASO assistance, highlighting notable improvements across all five systems.

Following this validation, we used the five ASO‐RCA systems to test a panel of 20 COVID‐19 positive and 20 negative patient saliva samples, previously classified by RT‐qPCR (*C*_t_ values provided in Table S3). For each test, RNA was cleaved for 30 min using the corresponding DNAzyme, followed by QE‐RCA with or without the addition of the system‐specific ASO. *F*_60_ was used to evaluate performance alongside positive and negative controls. Figure 5 summarizes the clinical testing results for all five systems

Panels a–e in Figure 5 show side‐by‐side comparisons of raw *F*_60_ values for each system with and without ASO assistance. For System 1, inclusion of ASO1 elevated signal intensities in nearly all positive samples while minimizing signal overlap with negatives, resulting in clearly improved separation. Cut‐off values (indicated by dotted lines) were determined based on receiver–operator characteristic (ROC) curve analyses, and the resulting clinical sensitivity, specificity, and accuracy values are displayed within each panel. System 1, for example, improved from 85% sensitivity and 50% specificity without ASO to 100% sensitivity and 80% specificity with ASO. Similar trends were observed across all systems, with particularly strong performance from Systems 3, 4, and 5, which achieved both 100% sensitivity and ≥95% specificity. Figures S17–S21 show box plots for all five systems, illustrating the maximum and minimum endpoint RFU values, interquartile ranges, and medians, as well as ROC curves comparing positive and negative samples, both with and without the corresponding ASOs.

Figure 5f summarizes the overall diagnostic accuracy across all five systems. Without ASOs, accuracy ranged from 67.5% (System 1) to 90% (Systems 2 and 5). In contrast, ASO‐assisted assays achieved accuracy values of 97.5%–100% for all systems, except for System 1, which improved to 90%—a substantial increase from the original 67.5% without ASO. These improvements are primarily due to strong signal enhancement in true positive samples while maintaining low background in true negatives, enabling the application of more stringent and reliable cut‐off thresholds. The detection of a sustained fluorescence signal after 60 min clearly demonstrates the stability of the assay components in clinical saliva samples.

It can be noted that some negative saliva samples exhibited an RCA signal both in the presence and absence of ASOs, particularly in Systems 1, 2, and 4. Although RT‐qPCR cycle threshold (*C*_t_) values for these negative samples are not available, it has been reported that minimally processed saliva can pose challenges for conventional RT‐PCR assays. This is primarily due to the physical characteristics of saliva (e.g., viscosity and heterogeneity) and the potential presence of unidentified inhibitory components. Notably, previous reports have shown that mixing saliva samples to disperse particulates prior to RT‐PCR can increase *C*_t_ values and lead to a higher incidence of false‐negative results, even in confirmed positive cases.^[^ ^58^ ^]^ These findings highlight the complexity of working with crude saliva and may partly explain the background signals observed in our study.

In summary, these results demonstrate that DNAzyme cleavage with ASO‐assisted RCA significantly enhances clinical performance by improving sensitivity, specificity, and diagnostic accuracy across diverse structured RNA targets. This highlights the robustness and broad applicability of ASOs for boosting amplification‐based molecular diagnostics in real‐world clinical samples.

## Conclusions

In summary, this study demonstrates that incorporating antisense oligonucleotides upstream of the circular DNA template binding site significantly enhances CDT hybridization to large structured RNAs, resulting in improved rolling circle amplification efficiency. Across five DNAzyme–RCA systems targeting SARS‐CoV‐2 genomic RNA, ASO inclusion led to substantial increases in fluorescence output—up to 70‐fold in complex biological matrices—and enabled effective amplification in both linear and quasi‐exponential RCA formats.

Importantly, ASO‐assisted RCA performed robustly in 50% pooled saliva and markedly improved diagnostic performance in clinical samples. For all five systems, ASOs enhanced signal separation between positive and negative samples, boosting clinical sensitivity to 100% and increasing diagnostic accuracy from as low as 67.5% to 90% in the most challenging case, and to 97.5%–100% in others. Table S4 presents a comparison between the newly developed assay and several recently reported RCA or saliva‐based molecular tests for SARS‐CoV‐2 detection, demonstrating the advantages of the new method in terms of assay temperature, assay time and overall clinical accuracy.^[^ ^59^, ^60^, ^61^, ^62^, ^63^, ^64^ ^]^

These results highlight ASO‐assisted RCA as a simple, cost‐effective, and broadly applicable strategy to overcome structural barriers in RNA targets. By facilitating more efficient primer–template hybridization, this approach enables more sensitive and reliable molecular diagnostics, offering strong potential for clinical translation in viral detection and beyond.

## Supporting Information

Comprehensive experimental procedures were placed in the Supporting Information.

## Author Contributions

Y.L., J.D.B., and A.M. designed the experiments and interpreted the data. A.M. performed all DNAzyme‐ coupled RCA with ASO experiments and analyzed the data. Y.L., J.D.B., and A.M. wrote the manuscript. All authors edited the manuscript.

## Conflict of Interests

The authors declare no conflict of interest.

## Supporting information



Supporting Information

## Data Availability

The data that support the findings of this study are available in the Supporting Information of this article.
