# Supplementary material for: Enhancing Clinical Detection Accuracy of Large Structured Viral RNA via DNAzyme Cleavage and Antisense‐Assisted Rolling Circle Amplification
Source: Angew Chem Int Ed Engl. 2025 Jun 26;64(33):e202507973. doi: 10.1002/anie.202507973 (PMC12338419; doi:10.1002/anie.202507973)
Supplement: Supplementary file 1 — Supporting Information [file ANIE-64-e202507973-s001.pdf]

Supplementary Information

Chemicals

Materials used in this study were sourced as follows:

Electrophoresis reagents, buffers and salts – Bioshop Canada or Millipore Sigma

Polynucleotide Kinase – Thermo Scientific

T4 DNA Ligase – Thermo Scientific

Q5 DNA Polymerase – New England Biolabs

T7 RNA Polymerase – Invitrogen

DNase I – Thermo Scientific

Pooled Human Saliva - Innovative Research

Proteinase K – Thermo Scientific

dNTPs & NTPs – Thermo Scientific

Phi29 DNA Polymerase – Thermo Scientific

RiboLock RNase Inhibitor – Thermo Scientific

SYBR Gold – Thermo Scientific

SYTO 9 – Thermo Scientific

TriTrack DNA loading Dye 6x – Thermo Scientific

FastDigest BamHI – Thermo Scientific

Oligonucleotide preparation

Oligonucleotides (linear RCA templates, antisense oligonucleotides, PCR primers) were obtained from Integrated DNA technologies and purified by standard 10% denaturing (8 M urea, 1X TBE) polyacrylamide gel electrophoresis (dPAGE) with the addition of 1X Loading Buffer (urea, 1 M Tris-HCl, 0.25 M EDTA, bromophenol blue, xylene cyanol), imaged by UV shadow and then excised. The product gel band was crushed, soaked in 1X elution buffer (200 mM NaCl, 10 mM Tris-HCl pH 7.5 and 1 mM EDTA pH 8) and shaken at room temperature for 20 minutes. EtOH precipitation was then performed by adding 2.5 volumes of chilled 100% EtOH to the elution buffer after the crushed gel fragments were removed through centrifugation. The EtOH-elution buffer mixture was chilled for 10 minutes at -20 °C to allow the oligo to aggregate and was precipitated by centrifugation for 20 minutes at 20,000 × g, followed by an additional 70% EtOH wash. The oligo pellet was then dried by SpeedVac, resuspended in nuclease-free H_2O, and spectrophotometrically quantified by A_{260} measurements using a DeNovix spectrophotometer. The sequences are listed in Table S1.

Circular DNA template (CDT) preparation:

PAGE purified linear RCA templates were phosphorylated at the 5' end using Adenosine Triphosphate (ATP) and Polynucleotide Kinase (PNK) in PNK Buffer A. After incubating the reaction for 20 minutes at 37 °C and heating at 90 °C for 5 minutes to deactivate the PNK, the linear strand was then circularized using ligation template, T4 DNA ligase and buffer. The assembled reaction was incubated at room temperature for 2 hours to allow circularization of the template. The CDT was purified on 10% dPAGE. CDT products were "double purified" by dPAGE (run on dPAGE gel twice) and quantified as described earlier.

RNA preparation

SARS-CoV-2 RNA gene of interest were transcribed from a SARS-CoV-2 DNA template, amplified by polymerase chain reaction (PCR) and purified using the New England Biolabs Monarch® PCR and DNA clean-up kit. Vectors for transcripts were kindly donated by Dr. Gerry Wright (McMaster University). Amplified and purified SARS-CoV-2 DNA was transcribed to RNA by 100 U T7 RNA Polymerase and incubated for 2 hours with 2 pmol of SARS-CoV-2 DNA, 5 mM dithiothreitol, 1× T7 RNA transcription buffer (20 mM TrisHCl pH 7.9, 3 mM $MgCl_2$, 5 mM DTT, 5 mM NaCl & 1 mM spermidine, Thermo Scientific™), 0.2 U pyrophosphatase, 2 mM of a nucleotide triphosphate mixture and 40 U Thermo Scientific RiboLock™, at 37 °C. Following this step, 5 U of DNase I was added and incubated for an additional 20 minutes at 37 °C. The transcribed SARS-CoV-2 RNA was then purified on 5% urea PAGE with the addition of 1X Quenching Buffer (10% sucrose, 0.5× TBE, 30 mM EDTA and 0.14 mg/mL bromophenol blue and xylene cyanol). The RNA band was then excised, crushed, soaked in 1X elution buffer, EtOH precipitated and dried by SpeedVac.

DNAzyme coupled RCA reactions

Coupled DNAzyme-RCA reactions were performed by assembling 50 nM DNAzyme, 10 nM RNA transcript, Reaction Buffer (0.5 M HEPES, 0.1 M $MgCl_2$, 1 M NaCl). Cleavage reactions were assembled without the RB. The reaction was initiated by addition of RB and incubated at 23 °C for 30 min. RCA reactions were setup using 50% DNAzyme reaction, 25 nM ASO, 10 nM circular RCA template, 250 μM dNTP, 2 μM Syto9, 0.25 U/μL PNK, 0.25 U/μL phi29 DNA Polymerase, phi29 reaction buffer (PB) containing 50 mM Tris pH 8.0, 10 mM $MgCl_2$, 66 mM KCl, 4 mM DTT, 0.1% Tween-20. Three control reactions were set up: CDT only (Negative control), CDT+ short RNA primer (Positive control), CDT+DZ+RNA (no ASO). CDT+DZ+RNA+ASO serves as the test reaction. Final reaction volumes were 20 μL. All reactions conducted in triplicate and incubated at 23 °C in a Bio-Rad CFX-96 real-time thermal cycler for 1 h while monitoring fluorescence using the FAM/SYBR filter.

RCA products on Agarose Gel

RCA reaction products were divided into two aliquots: one untreated (−Digest) and one subjected to restriction digestion (+Digest). For the +Digest samples, 1 μL of Thermo FastDigest BamHI was added, and the mixture was incubated at 37 °C for 5 minutes. Subsequently, 3 μL of 6X agarose loading dye was added to each reaction. A total of 10 μL from each sample was loaded onto a 1.5% TBE agarose gel containing SybrSafe DNA stain. Additionally, 2 μL each of low-range and high-range DNA ladders were loaded. Electrophoresis was carried out at 120 V for approximately 40 minutes, or until the loading dye migrated to the middle of the gel. Gels were imaged using a Cy2 filter on a Typhoon scanner.

Native Gel Shift Assay

2 pmol of short 63 nt RNA transcript along with PAGE purified ASOs (80 nt) and 1X RB were incubated at room temperature for 10 minutes to facilitate hybridization between RNA and respective ASOs. The reactions, with the addition of 1X DNA loading dye, were added to a 10% Native PAGE gel which ran for 4 hours at 350 Volts. The gel was then stained with SYBR gold and imaged on Amersham™ Typhoon 9200 scanner with a Cy2 filter.

Saliva processing and clinical validation experiments

Saliva specimens collected using protocols approved by the Hamilton Integrated Research Ethics Board (HiREB Project # 12636). Patients visiting COVID-19 assessment centers operated by Hamilton Health Sciences or St. Joseph's Healthcare in Hamilton, Ontario, were asked to provide a supervised, self-collected saliva sample (drool) immediately after a nasopharyngeal swab (NPS). Saliva samples were stored at 4 °C during transport (up to 72 hours) and then preserved long-term at -80 °C. NPS samples, collected for routine COVID-19 screening, were analyzed using a standard RT-PCR method by the Hamilton Regional Laboratory Medicine Program at St. Joseph's Healthcare. NPS test results were used to identify 20 negative (NS1-20) and 20 positive (PS1-20) saliva samples, and to determine the putative variants associated with positive cases. The presence of SARS-CoV-2 in the selected saliva samples was further validated using a previously described saliva RT-PCR method.[1,2] 20 positive and 20 negative human pooled donor saliva samples were treated by addition of 2.5 mg/mL Proteinase K, 10 mM Tris pH 8.0, 1 mM EDTA. Samples were incubated at 23 °C for 2 min and then heated at 90 °C for 10 min. Cleavage

reactions performed with 50 nM DNAzyme and RB. Negative control reaction setup with buffer, and positive control reactions with DNAzyme, 25 nM ASO and 5 nM RNA transcript. RCA reactions setup as mentioned in DNAzyme-coupled RCA methods with the addition of P2 primer for quasi-exponential RCA. All reactions conducted in triplicate and incubated at 23 °C in a Bio-Rad CFX-96 real-time thermal cycler for 1 h while monitoring fluorescence using the FAM/SYBR filter.

Table S1. Sequences of all relevant DNA and RNA sequences.

Oligonucleotides	Length (nucleotides)	Sequence (5' - 3')
**RNA**		
ls584	584	GGGCCUCAGAGUUUAGUUCCCUUCCAUCAUAUGCAGCUUUUGCUACUGCUCAAGAAG CUUAUGAGCAGGCUGUUGCUAAUGGUGAUUCUGAAGUUGUUCUUAAAAAGUUGAAGAA GUCUUUGAAUGUGGCUAAAUCUGAAUUUGACCGUGAUGCAGCCAUGCAACGUAAGUU GGAAAAGAUGGCUGAUCAAGCUAUGACCCAAAUGUAUAAACAGGCUAGAUCUGAGGAC AAGAGGGCAAAAGUUACUAGUGCUAUGCAGACAAUGCUUUUCACUAUGCUUAGAAAGU UGGAUAAUGAUGCACUCAACAACAUUAUCAACAAUGCAAGAGAUGGUUGUGUUCCCUU GAACAUAAUACCUCUUACAACAGCAGCCAAACUAAUGGUUGUCAUACCAGACUAUAACA CAUAUAAAAAUACGUGUGAUGGUACAACAUUUACUUAUGCAUCAGCAUUGUGGGAAAU CCAACAGGUUGUAGAUGCAGAUAGUAAAAUUGUUCAACUUAGUGAAAUUAGUAUGGAC AAUUCACCUAAUUUAGCAUGGCCUCUUAUUGUAACAGCUUUAAGGGCCAAUUCUGCUG UCAAA
ls1798	1798	GGGCUGGUGAGUUUAAAUUGGCUUCACAUAUGUAUUGUUCUUUCUACCCUCCAGAUG AGGAUGAAGAAGAAGGUGAUUGUGAAGAAGAAGAGUUUGAGCCAUCAACUCAAUAUGA GUAUGGUACUGAAGAUGAUUACCAAGGUAAACCUUUGGAAUUUGGUGCCACUUCUGC UGCUCUUCAACCUGAAGAAGAGCAAGAAGAAGAUUGGUUAGAUGAUGAUAGUCAACAA ACUGUUGGUCAACAAGACGGCAGUGAGGACAAUCAGACAACUACUAUUCAAACAAUUG UUGAGGUUCAACCUCAAUUAGAGAUGGAACUUACACCAGUUGUUCAGACUAUUGAAGU GAAUAGUUUUAGUGGUUAUUUAAAACUUACUGACAAUGUAUACAUUAAAAAUGCAGACA UUGUGGAAGAAGCUAAAAAGGUAAAACCAACAGUGGUUGUUAAUGCAGCCAAUGUUUA CCUUAAACAUGGAGGAGGUGUUGCAGGAGCCUUAAAUAAGGCUACUAACAAUGCCAUG CAAGUUGAAUCUGAUGAUUACAUAGCUACUAAUGGACCACUUAAAGUGGGUGGUAGUU GUGUUUUAAGCGGACACAAUCUUGCUAAACACUGUCUUCAUGUUGUCGGCCCAAAUGU UAACAAAGGUGAAGACAUUCAACUUCUUAAGAGUGCUUAUGAAAAUUUUAAUCAGCAC GAAGUUCUACUUGCACCAUUAUUAUCAGCUGGUAUUUUUGGUGCUGACCCUAUACAUU CUUUAAGAGUUUGUGUAGAUACUGUUCGCACAAAUGUCUACUUAGCUGUCUUUGAUAA AAAUCUCUAUGACAAACUUGUUUCAAGCUUUUUGGAAAUGAAGAGUGAAAAGCAAGUU GAACAAAAGAUCGCUGAGAUUCCUAAAGAGGAAGUUAAGCCAUUUAUAACUGAAAGUA AACCUUCAGUUGAACAGAGAAAACAAGAUGAUAAGAAAAUCAAAGCUUGUGUUGAAGAA

		GUUACAACAACUCUGGAAGAAACUAAGUUCCUCACAGAAAACUUGUUACUUUAUAUUG<br>ACAUUAAUGGCAAUCUUCAUCCAGAUUCUGCCACUCUUGUUAGUGACAUUGACAUCAC<br>UUUCUUAAAGAAAGAUGCUCCAUAUAUAGUGGGUGAUGUUGUUCAAGAGGGUGUUUU<br>AACUGCUGUGGUUAUACCUACUAAAAAGGCUGGUGGCACUACUGAAAUGCUAGCGAAA<br>GCUUUGAGAAAAGUGCCAACAGACAAUUAUAUAACCACUUACCCGGGUCAGGGUUUAA<br>AUGGUUACACUGUAGAGGAGGCAAAGACAGUGCUUAAAAAGUGUAAAAGUGCCUUUUA<br>CAUUCUACCAUCUAUUAUCUCUAAUGAGAAGCAAGAAAUUCUUGGAACUGUUUCUUGG<br>AAUUUGCGAGAAAUGCUUGCACAUGCAGAAGAAACACGCAAAUUAAUGCCUGUCUGUG<br>UGGAAACUAAAGCCAUAGUUUCAACUAUACAGCGUAAAUAUAAGGGUAUUAAAAUACAA<br>GAGGGUGUGGUUGAUUAUGGUGCUAGAUUUUACUUUUACACCAGUAAAACAACUGUA<br>GCGUCACUUAUCAACACACUUAACGAUCUAAAUGAAACUCUUGUUACAAUGCCACUUG<br>GCUAUGUAACACAUGGCUUAAAUUUGGAAGAAGCUGCUCGGUAUAUGAGAUCUCUCAA<br>AGUGCCAGCUACAGUUUCUGUUUCUUCACCUGAUGCUGUUACAGCGUAUAAUGGUUA<br>UCUUACUUCUUCUUCUAAAACACCUGAAGAACAUUUUAUUGAAACCAUCUCACUUGCU<br>GG
ls1208	1208	GGGUUUGCGGUGUAAGUGCAGCCCGUCUUACACCGUGCGGCACAGGCACUAGUACUG<br>AUGUCGUAUACAGGGCUUUUGACAUCUACAAUGAUAAAGUAGCUGGUUUUGCUAAAUU<br>CCUAAAAACUAAUUGUUGUCGCUUCCAAGAAAAGGACGAAGAUGACAAUUUAAUUGAU<br>UCUUACUUUGUAGUUAAGAGACACACUUUCUCUAACUACCAACAUGAAGAAACAAUUUA<br>UAAUUUACUUAAGGAUUGUCCAGCUGUUGCUAAACAUGACUUCUUUAAGUUUAGAAUA<br>GACGGUGACAUGGUACCACAUAUAUCACGUCAACGUCUUACUAAAUACACAAUGGCAG<br>ACCUCGUCUAUGCUUUAAGGCAUUUUGAUGAAGGUAAUUGUGACACAUUAAAAGAAAU<br>ACUUGUCACAUACAAUUGUUGUGAUGAUGAUUAUUUCAAUAAAAAGGACUGGUAUGAU<br>UUUGUAGAAAACCCAGAUAUAUUACGCGUAUACGCCAACUU<br>AGGUGAACGUGUACGCCAAGCUUUGUUAAAAACAGUACAAUUCUGUGAUGCCAUGCGA<br>AAUGCUGGUAUUGUUGGUGUACUGACAUUAGAUAAUCAAGAUCUCAAUGGUAACUGGU<br>AUGAUUUCGGUGAUUUCAUACAAACCACGCCAGGUAGUGGAGUUCCUGUUGUAGAUU<br>CUUAUUAUUCAUUGUUAAUGCCUAUAUUAACCUUGACCAGGGCUUUAACUGCAGAGUC<br>ACAUGUUGACACUGACUUAACAAAGCCUUACAUUAAGUGGGAUUUGUUAAAAUAUGAC<br>UUCACGGAAGAGAGGUUAAAACUCUUUGACCGUUAUUUUAAAUAUUGGGAUCAGACAU<br>ACCACCCAAAUUGUGUUAACUGUUUGGAUGACAGAUGCAUUCUGCAUUGUGCAAACUU<br>UAAUGUUUUAUUCUCUACAGUGUUCCCACCUACAAGUUUUGGACCACUAGUGAGAAAA<br>AUAUUUGUUGAUGGUGUUCCAUUUGUAGUUUCAACUGGA<br>UACCACUUCAGAGAGCUAGGUGUUGUACAUAAUCAGGAUGUAAACUUACAUAGCUCUA<br>GACUUAGUUUUAAGGAAUUACUUGUGUAUGCUGCUGACCCUGCUAUGCACGCUGCUU

		CUGGUAAUCUAUUACUAGAUAAACGCACUACGUGCUUUUCAGUAGCUGCACUUACUAA<br>CAAUGUUGCUUUUCAAACUGUCAAACCC
ls1805	1805	GGGCUGUUGGGGCUUGUGUUCUUUGCAAUUCACAGACUUCAUUAAGAUGUGGUGCUU<br>GCAUACGUAGACCAUUCUUAUGUUGUAAAUGCUGUUACGACCAUGUCAUAUCAACAUC<br>ACAUAAAUUAGUCUUGUCUGUUAAUCCGUAUGUUUGCAAUGCUCCAGGUUGUGAUGU<br>CACAGAUGUGACUCAACUUUACUUAGGAGGUAUGAGCUAUUAUUGUAAAUCACAUAAA<br>CCACCCAUUAGUUUUCCAUUGUGUGCUAAUGGACAAGUUUUUGGUUUAUAUAAAAAUA<br>CAUGUGUUGGUAGCGAUAAUGUUACUGACUUUAAUGCAAUUGCAACAUGUGACUGGAC<br>AAAUGCUGGUGAUUACAUUUUAGCUAACACCUGUACUGAAAGACUCAAGCUUUUUGCA<br>GCAGAAACGCUCAAAGCUACUGAGGAGACAUUUAAACUGUCUUAUGGUAUUGCUACUG<br>UACGUGAAGUGCUGUCUGACAGAGAAUUACAUCUUUCAUGGGAAGUUGGUAAACCUAG<br>ACCACCACUUAACCGAAAUUAUGUCUUUACUGGUUAUCGUGUAACUAAAAACAGUAAA<br>GUACAAAUAGGAGAGUACACCUUUGAAAAAGGUGACUAUGGUGAUGCUGUUGUUUACC<br>GAGGUACAACAACUUACAAAUUAAAUGUUGGUGAUUAUUUUGUGCUGACAUCACAUAC<br>AGUAAUGCCAUUAAGUGCACCUACACUAGUGCCACAAGAGCACUAUGUUAGAAUUACU<br>GGCUUAUACCCAACACUCAAUAUCUCAGAUGAGUUUUCUAGCAAUGUUGCAAAUUAUC<br>AAAAGGUUGGUAUGCAAAAGUAUUCUACACUCCAGGGACCACCUGGUACUGGUAAGAG<br>UCAUUUUGCUAUUGGCCUAGCUCUCUACUACCCUUCUGCUCGCAUAGUGUAUACAGC<br>UUGCUCUCAUGCCGCUGUUGAUGCACUAUGUGAGAAGGCAUUAAAAUAUUUGCCUAUA<br>GAUAAAUGUAGUAGAAUUAUACCUGCACGUGCUCGUGUAGAGUGUUUUGAUAAAUUCA<br>AAGUGAAUUCAACAUUAGAACAGUAUGUCUUUUGUACUGUAAAUGCAUUGCCUGAGAC<br>GACAGCAGAUAUAGUUGUCUUUGAUGAAAUUUCAAUGGCCACAAAUUAUGAUUUGAGU<br>GUUGUCAAUGCCAGAUUACGUGCUAAGCACUAUGUGUACAUUGGCGACCCUGCUCAA<br>UUACCUGCACCACGCACAUUGCUAACUAAGGGCACACUAGAACCAGAAUAUUUCAAUU<br>CAGUGUGUAGACUUAUGAAAACUAUAGGUCCAGACAUGUUCCUCGGAACUUGUCGGC<br>GUUGUCCUGCUGAAAUUGUUGACACUGUGAGUGCUUUGGUUUAUGAUAAUAAGCUUA<br>AAGCACAUAAAGACAAAUCAGCUCAAUGCUUUAAAAUGUUUUAUAAGGGUGUUAUCAC<br>GCAUGAUGUUUCAUCUGCAAUUAACAGGCCACAAAUAGGCGUGGUAAGAGAAUUCCUU<br>ACACGUAACCCUGCUUGGAGAAAAGCUGUCUUUAUUUCACCUUAUAAUUCACAGAAUG<br>CUGUAGCCUCAAAGAUUUUGGGACUACCAACUCAAACUGUUGAUUCAUCACAGGGCUC<br>AGAAUAUGACUAUGUCAUAUUCACUCAAACCACUGAAACAGCUCACUCUUGUAAUGUAA<br>ACAGAUUUAAUGUUGCUAUUACCAGAGCAAAAGUAGGCAUACUUUGCAUAAUGUCUGA<br>UAGAGACCUUUAUGACAAGUUGCAAUUUACAAGUCUUGAAAUUCCACGUAGGAAUGUG<br>GCAACUUUACAA

ls831	831	GGGAUGGAUUUGUUUAUGAGAAUCUUCACAAUUGGAACUGUAACUUUGAAGCAAGGU<br>GAAAUCAAGGAUGCUACUCCUUCAGAUUUUGUUCGCGCUACUGCAACGAUACCGAUAC<br>AAGCCUCACUCCCUUUCGGAUGGCUUAUUGUUGGCGUUGCACUUCUUGCUGUUUUUC<br>AGAGCGCUUCCAAAAUCAUAACCCUCAAAAAGAGAUGGCAACUAGCACUCUCCAAGGG<br>UGUUCACUUUGUUUGCAACUUGCUGUUGUUGUUUGUAACAGUUUACUCACACCUUUU<br>GCUCGUUGCUGCUGGCCUUGAAGCCCCUUUUCUCUAUCUUUAUGCUUUAGUCUACUU<br>CUUGCAGAUAUAAACUUUGUAAGAAUAAUAAUGAGGCUUUGGCUUUGCUGGAAAUGCC<br>GUUCCAAAAACCCAUUACUUUAUGAUGCCAACUAUUUUCUUUGCUGGCAUACUAAUUG<br>UUACGACUAUUGUAUACCUUACAAUAGUGUAACUUCUUCAAUUGUCAUUACUUCAGGU<br>GAUGGCACAACAAGUCCUAUUUCUGAACAUGACUACCAGAUUGGUGGUUAUACUGAAA<br>AAUGGGAAUCUGGAGUAAAAGACUGUGUUGUAUUACACAGUUACUUCACUUCAGACUA<br>UUACCAGCUGUACUCAACUCAAUUGAGUACAGACACUGGUGUUGAACAUGUUACCUUC<br>UUCAUCUACAAUAAAAUUGUUGAUGAGCCUGAAGAACAUGUCCAAAUUCACACAAUCGA<br>CGGUUCAUCCGGAGUUGUUAAUCCAGUAAUGGAACCAAUUUAUGAUGAACCGACGACG<br>ACUACUAGCGUGCCUUUGUAA
63nt RNA1	63	AAUCCAACAGGUUGUAGAUGCAGAUAGUAAAAUUGUUCAACUUAGUGAAAUUAGUAUG<br>GACAA
63nt RNA2	63	UUGACAUCACUUUCUUAAAGAAAGAUGCUCCAUAUAUAGUGGGUGAUGUUGUUCAAGA<br>GGGUG
63nt RNA3	63	ACACUUUCUCUAACUACCAACAUGAAGAAACAAUUUAUAAUUUACUUAAGGAUUGUCCA<br>GCUG
63nt RNA4	63	CACGCACAUUGCUAACUAAGGGCACACUAGAACCAGAAUAUUUCAAUUCAGUGUGUAG<br>ACUUA
63nt RNA5	63	AUAAACUUUGUAAGAAUAAUAAUGAGGCUUUGGCUUUGCUGGAAAUGCCGUUCCAAAA<br>ACCCA
**DNA**		
CDT1	51	TCCCCATTTATTTGTCCATACTAATTTCACTGTTAGAGTTTTTCATTAGGA
CDT2	51	TCCCCATTTATCACCCTCTTGAACAACATCAGTTAGAGTTTTTCATTAGGA

CDT3	51	TCCCCATTTATCAGCTGGACAATCCTTAAGTGTTAGAGTTTTTCATTAGGA
CDT4	51	TCCCCATTTATTAAGTCTACACACTGAATTGGTTAGAGTTTTTCATTAGGA
CDT5	51	TCCCCATTTATTGGGTTTTTGGAACGGCATTGTTAGAGTTTTTCATTAGGA
dz_12618a	39	TGCTAAATTAGGTGAAGGCTAGCTACAACGATGTCCATA
dz_25806a	39	TGGCATCATAAAGTAAGGCTAGCTACAACGAGGGTTTTT
dz_13726a	39	AAGTCATGTTTAGCAAGGCTAGCTACAACGAAGCTGGAC
dz_17522a	39	GGACCTATAGTTTTCAGGCTAGCTACAACGAAAGTCTAC
dz_4148a	39	ACCACAGCAGTTAAAAGGCTAGCTACAACGAACCCTCTT
ASO1	80	TTGAACAATTTTACTATCTGCATCTACAACCTGTTGGATTTCCCACAATGCTGATGCATAAGT AAATGTTGTACCATCAC
ASO2	80	ACTATATATGGAGCATCTTTCTTTAAGAAAGTGATGTCAATGTCACTAACAAGAGTGGCAG AATCTGGATGAAGATTGCC
ASO3	80	TTATAAATTGTTTCTTCATGTTGGTAGTTAGAGAAAGTGTGTCTCTTAACTACAAAGTAAGAA TCAATTAAATTGTCATCT
ASO4	80	TATTCTGGTTCTAGTGTGCCCTTAGTTAGCAATGTGCGTGGTGCAGGTAATTGAGCAGGGT CGCCAATGTACACATAGTG
ASO5	80	GAAGGAGTAGCATCCTTGATTTCACCTTGCTTCAAAGTTACAGTTCCAATTGTGAAGATTC TCATAAACAAATCCATCCC

Table S2. k_{obs} values and coupling category of the selected 5 coupling systems.

System	k_{obs} (min^{-1})	Coupling category
Is584/dZ_12618a/CDT1	0.027	Good
Is1798/dZ_4148a/CDT2	0.19	Good
Is1208/dZ_13726a/CDT3	0.094	Good
Is1805/dZ_17522a/CDT4	0.12	Poor
Is831/dZ_25806a/CDT5	0.09	Poor

Table S3. Clinical patient sample SARS-CoV-2 test results for nasopharyngeal samples (NPS) and saliva samples. NPS samples were tested at Hamilton Regional Medical Laboratory Program. Samples were assigned a presumed variant based on epidemiological context at the time of sample collection, indicative RT-PCR results, or if confirmed by sequencing. Coupled DNAzyme-RCA-ASO results were performed in triplicate using saliva, the mean RFU values are reported at 1 h reaction time.

ID	SARS-CoV-2 Status	Ct Value (HRLMP)				Variant	Saliva Ct value N1 target	DNAzyme-RCA-ASO mean RFU				
		UTR	E	ORF	N			System 1	System 2	System 3	System 4	System 5
PS1	POSITIVE	0	0	17.38	15.1	B.1.1.529	25.49	5609	42306	38754	19200	15576
PS2	POSITIVE	20.97	18.59	0	0	BA.5	27.41	12949	42183	47027	15061	15890
PS3	POSITIVE	0	0	17.33	14.51	B.1.1.529	27.5	13005	19934	38090	45320	15788
PS4	POSITIVE	14.67	12.31	0	0	BA.2.12.1	36.82	10729	32885	45519	18236	14027
PS5	POSITIVE	16.05	14.22	0	0		27.35	23944	40067	43101	24212	13398
PS6	POSITIVE	15.39	13.28	0	0		25.04	7441	38090	46402	30612	16705
PS7	POSITIVE	14.4	12.4	0	0		28.13	13044	38637	44185	16631	9960
PS8	POSITIVE	16.56	14.89	0	0	BA.4	29.59	21693	40581	40841	42582	10002
PS9	POSITIVE	0	0	15.13	12.79	B.1.1.529	30.33	36563	36621	46860	47713	9901
PS10	POSITIVE	12.38	10.82	0	0	BA.2.12.1	34.79	34022	29974	44803	37177	10432
PS11	POSITIVE	0	0	27.4	24.46	B.1.1.529	24.62	22338	38123	48786	46840	10940
PS12	POSITIVE	0	0	16.78	12.54	B.1.1.529	15.46	21792	28222	41748	49173	9559
PS13	POSITIVE	18.22	16.01	0	0	BA.5	26.3	16474	33428	50294	45130	17431
PS14	POSITIVE	15.25	13.13	0	0	BA.5	34.16	10668	34936	41834	47632	15974
PS15	POSITIVE	16.3	11.25	0	0	BA.4	37.3	20368	28492	46782	41435	16029
PS16	POSITIVE	23.56	17.23	0	0	BA.2.12.1	21.45	17496	30282	41850	2162	17863
PS17	POSITIVE	15.71	14.11	0	0	BA.2.12.1	30.3	24110	27915	43224	34131	17467
PS18	POSITIVE	15.08	13.38	0	0	BA.5	28.77	16378	29324	41145	17201	16966
PS19	POSITIVE	18.59	13.01	0	0	BA.5	22.84	16214	20427	41683	22321	17429
PS20	POSITIVE	24.53	18	0	0	BA.2.12.1	24.03	49888	19811	44652	14403	18545
NS1	NEGATIVE							2885	5155	3470	13036	782
NS2	NEGATIVE							3403	4975	6821	1580	1154
NS3	NEGATIVE							3082	10367	5728	1204	1238
NS4	NEGATIVE							1386	5063	9704	1362	1117
NS5	NEGATIVE							2304	13397	2790	1812	1238
NS6	NEGATIVE							2597	5275	3317	1477	1115
NS7	NEGATIVE							10523	11379	2813	11412	1360
NS8	NEGATIVE							688	4480	2046	1811	714
NS9	NEGATIVE							2912	3311	2245	4175	705
NS10	NEGATIVE							8572	3849	1766	11437	1753
NS11	NEGATIVE							776	3084	667	1116	1222
NS12	NEGATIVE							1441	3195	651	1150	1450
NS13	NEGATIVE							1138	15021	550	18111	1308
NS14	NEGATIVE							1097	4217	4379	1318	1262
NS15	NEGATIVE							767	4560	5661	1383	961
NS16	NEGATIVE							1211	5727	4445	7511	528
NS17	NEGATIVE							14049	5347	10500	1265	341
NS18	NEGATIVE							627	5095	11428	1194	278
NS19	NEGATIVE							718	11544	4875	1365	363
NS20	NEGATIVE							7169	2412	12590	1170	466

Table S4. A summary of RCA and/or saliva-based molecular tests developed for SARS-CoV-2 detection. Approximate test times are provided and include saliva sample preparation, device setup, and reaction time. The specific viral gene targets used in each assay are also listed.

Technology	Technical requirements	Target	Samples tested	Test time	Sensitivity	Specificity	Ref
ASO-RCA	23 °C	Any target	Saliva	1 hr 45 min	100%	100%	This test
RT-qPCR	Thermal cycler	ORF1ab/ORF8/ RdRp / E / N / S	NPS	2-24 hr	50% - 96%	70.4%- 100%	[59]
RT- Loop-mediated Isothermal Amplification (LAMP)	65 °C	ORF1ab/ORF7a/S	Saliva and NPS	1 hr	83%	100%	[60]
Recombinase Polymerase Amplification (RPA)	42 °C	S	Spiked saliva samples	2 hr	100%	100%	[61]
Multi-region padlock hybridization RCA (MP-RCA)	37 °C	ORF/RdRp/E/N/S	NPS	3 hr	81.8%	88%	[62]
Netlike RCA (NRCA)	30 °C	RdRp	SARS-CoV-2 RNA transcript	4 hr	-	-	[63]
AND-Logic Cascade RCA	37 °C	N	NPS	2.5 hr	88.2%	-	[64]

NSP = non structured protein, ORF = open reading frame, RdRp = RNA-dependent RNA polymerase, E = envelope, N = nucleocapsid, S = spike.

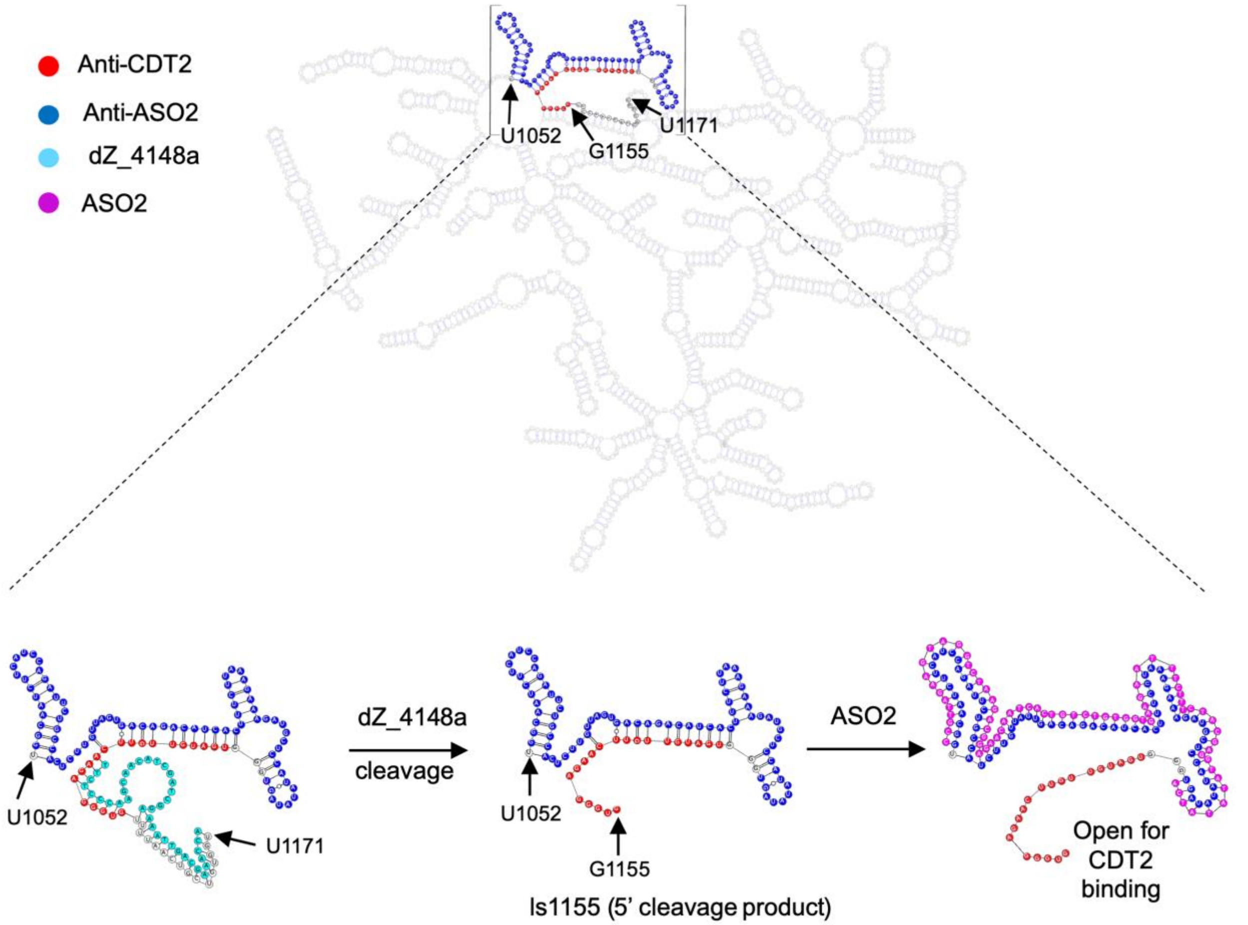

Figure S1. Secondary structure analysis of the ls1798 RNA transcript (System 2) and the mechanism by which ASO2 enhances accessibility to the CDT2-binding region. The full secondary structure of ls1798, predicted using the Mathews Lab RNAstructure software, is shown at the top, with the CDT2-binding region (Anti-CDT2) in red and the ASO2-binding region (Anti-ASO2) in blue. The bottom-left structure shows the DNAzyme dZ_4148a (shown in cyan) bound to ls1798, cleaving the RNA between G1155 and U1156. The resulting 5′-cleavage product (bottom center) retains significant secondary structure, with the Anti-CDT2 region (red) still partially sequestered. Upon addition of ASO2 (magenta, bottom right), the upstream RNA structure is reorganized, freeing the Anti-CDT2 region and enabling effective hybridization with the circular DNA template (CDT2) for RCA initiation.

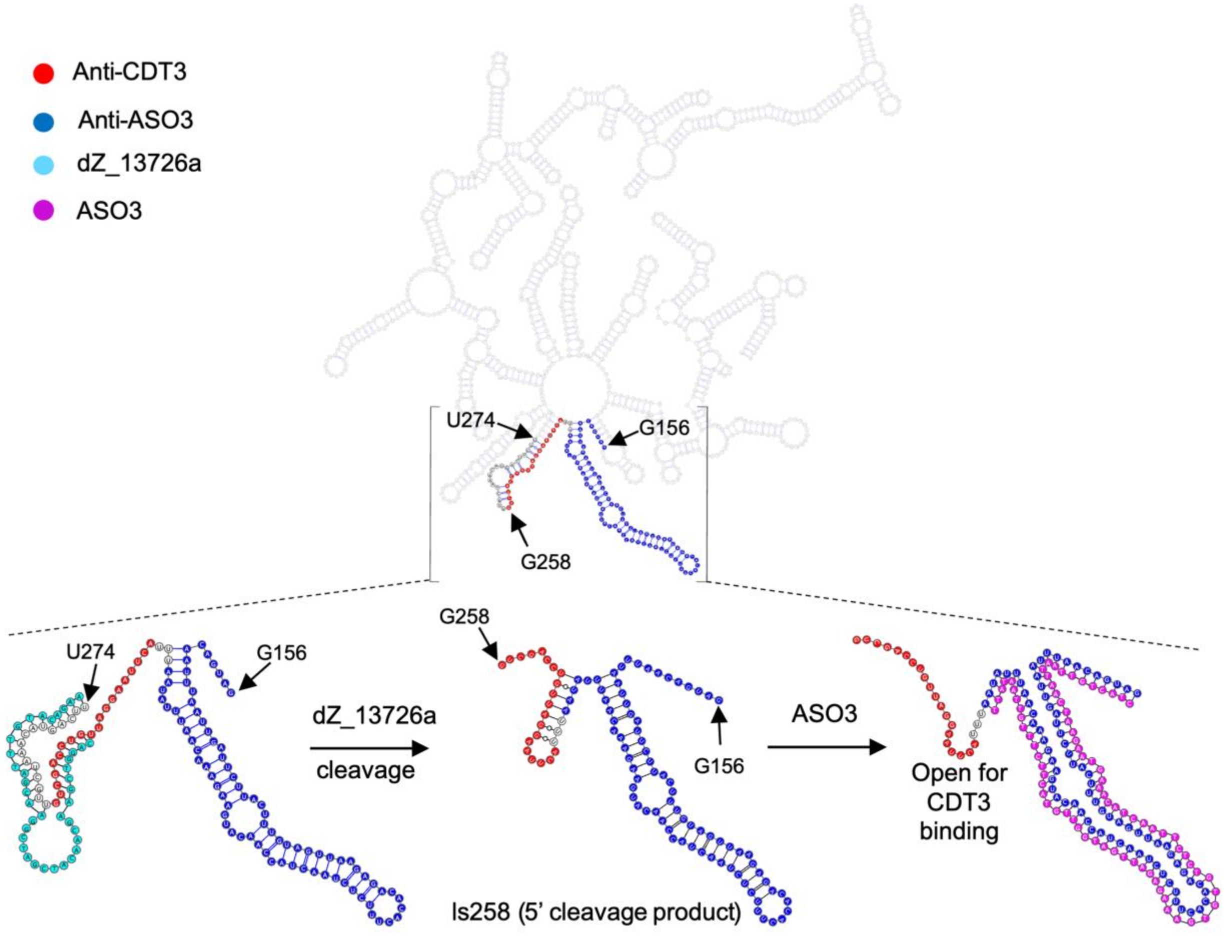

Figure S2. Secondary structure analysis of the ls1208 RNA transcript (System 3) and the mechanism by which ASO3 enhances accessibility to the CDT3-binding region. The full secondary structure of ls1208, predicted using the Mathews Lab RNAstructure software, is shown at the top, with the CDT3-binding region (Anti-CDT3) in red and the ASO3-binding region (Anti-ASO3) in blue. The bottom-left structure shows the DNAzyme dZ_13726a (shown in cyan) bound to ls13726, cleaving the RNA between G258 and U259. The resulting 5′-cleavage product (bottom center) retains significant secondary structure, with the Anti-CDT3 region (red) still partially sequestered. Upon addition of ASO3 (magenta, bottom right), the upstream RNA structure is reorganized, freeing the Anti-CDT3 region and enabling effective hybridization with the circular DNA template (CDT3) for RCA initiation.

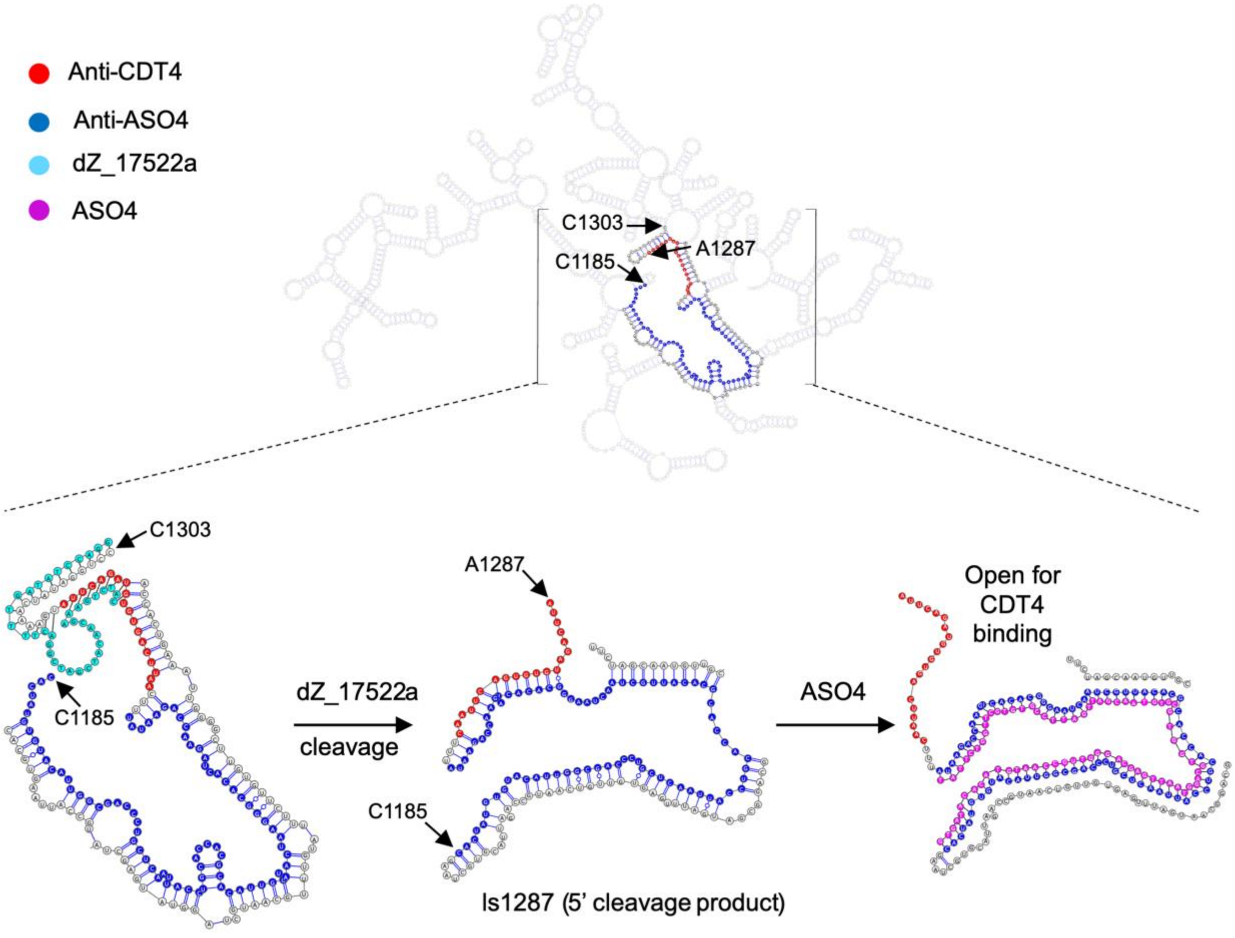

Figure S3. Secondary structure analysis of the ls1805 RNA transcript (System 4) and the mechanism by which ASO4 enhances accessibility to the CDT4-binding region. The full secondary structure of ls1805, predicted using the Mathews Lab RNAstructure software, is shown at the top, with the CDT4-binding region (Anti-CDT4) in red and the ASO4-binding region (Anti-ASO4) in blue. The bottom-left structure shows the DNAzyme dZ_17522a (shown in cyan) bound to ls1805, cleaving the RNA between A1287 and U1288. The resulting 5′-cleavage product (bottom center) retains significant secondary structure, with the Anti-CDT4 region (red) still partially sequestered. Upon addition of ASO4 (magenta, bottom right), the upstream RNA structure is reorganized, freeing the Anti-CDT4 region and enabling effective hybridization with the circular DNA template (CDT4) for RCA initiation.

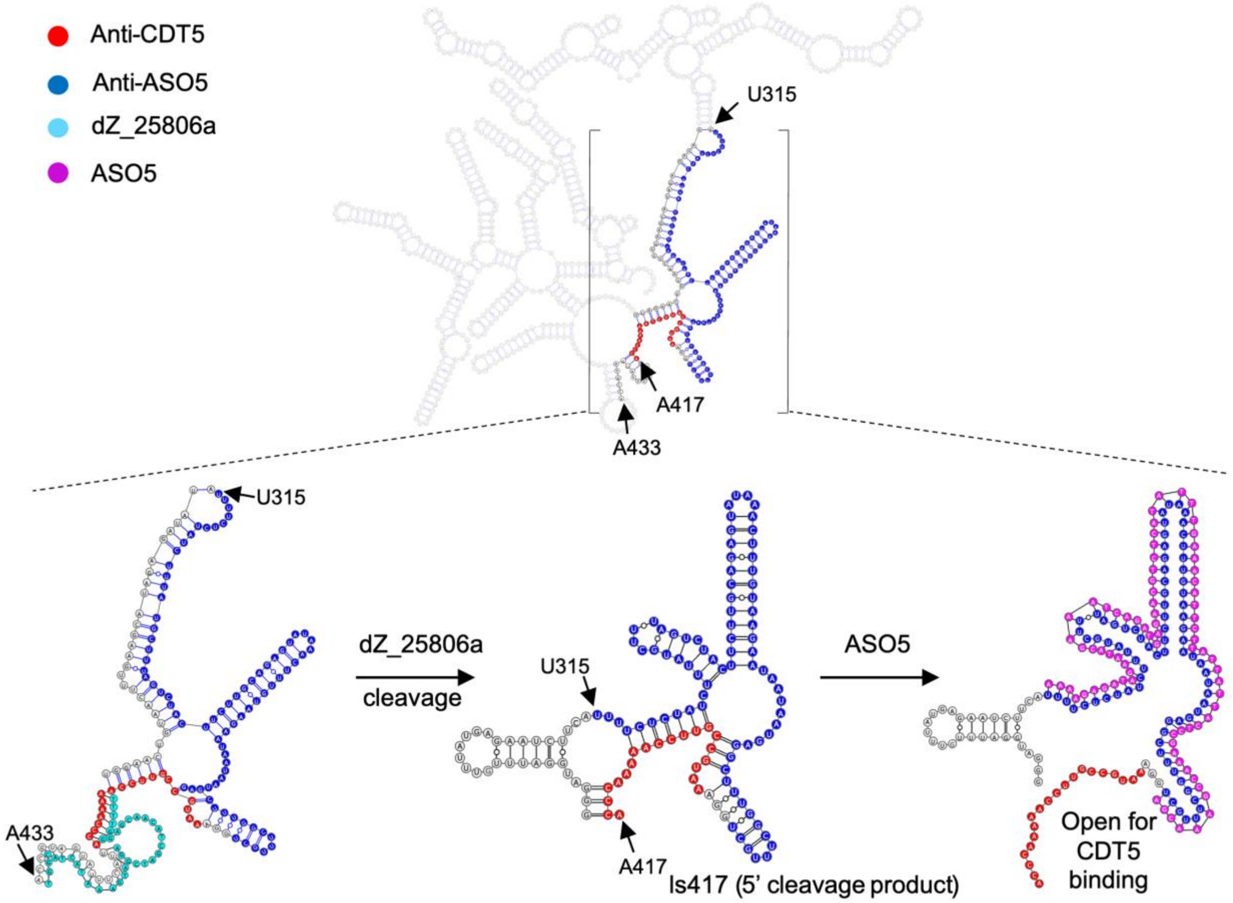

Figure S4. Secondary structure analysis of the ls831 RNA transcript (System 5) and the mechanism by which ASO5 enhances accessibility to the CDT5-binding region. The full secondary structure of ls831, predicted using the Mathews Lab RNAstructure software, is shown at the top, with the CDT5-binding region (Anti-CDT5) in red and the ASO5-binding region (Anti-ASO5) in blue. The bottom-left structure shows the DNAzyme dZ_25806a (shown in cyan) bound to ls831, cleaving the RNA between A417 and U418. The resulting 5′-cleavage product (bottom center) retains significant secondary structure, with the Anti-CDT5 region (red) still partially sequestered. Upon addition of ASO5 (magenta, bottom right), the upstream RNA structure is reorganized, freeing the Anti-CDT5 region and enabling effective hybridization with the circular DNA template (CDT5) for RCA initiation.

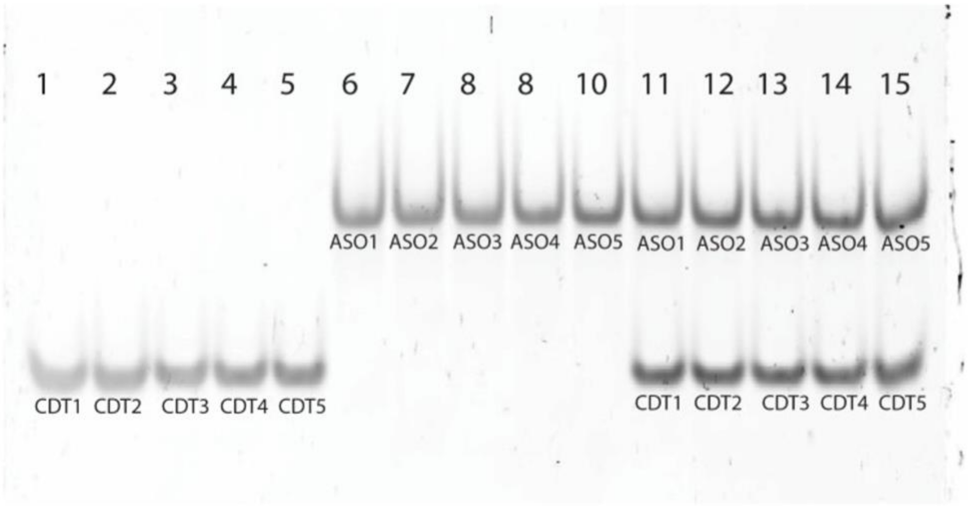

Figure S5. Native gel shift assay demonstrating that ASOs did not bind their corresponding CDTs, confirming that ASOs do not act as primers or form nonspecific complexes with circular DNA templates (CDTs). Lanes 1–5 show individual migration of CDT1 through CDT5, respectively, while lanes 6–10 show ASO1 through ASO5 alone. Lanes 11–15 contain mixtures of each CDT with its respective ASO (e.g., CDT1 + ASO1, CDT2 + ASO2, etc.). In all cases, no mobility shift was observed upon mixing, and the migration patterns remained identical to those of the individual components. These results indicate that ASOs do not hybridize to CDTs under the assay conditions, ruling out the possibility of nonspecific ASO priming or interactions that could lead to background RCA. This supports the conclusion that ASO-enhanced RCA efficiency is due specifically to ASO-mediated restructuring of RNA, not direct interactions with CDT.

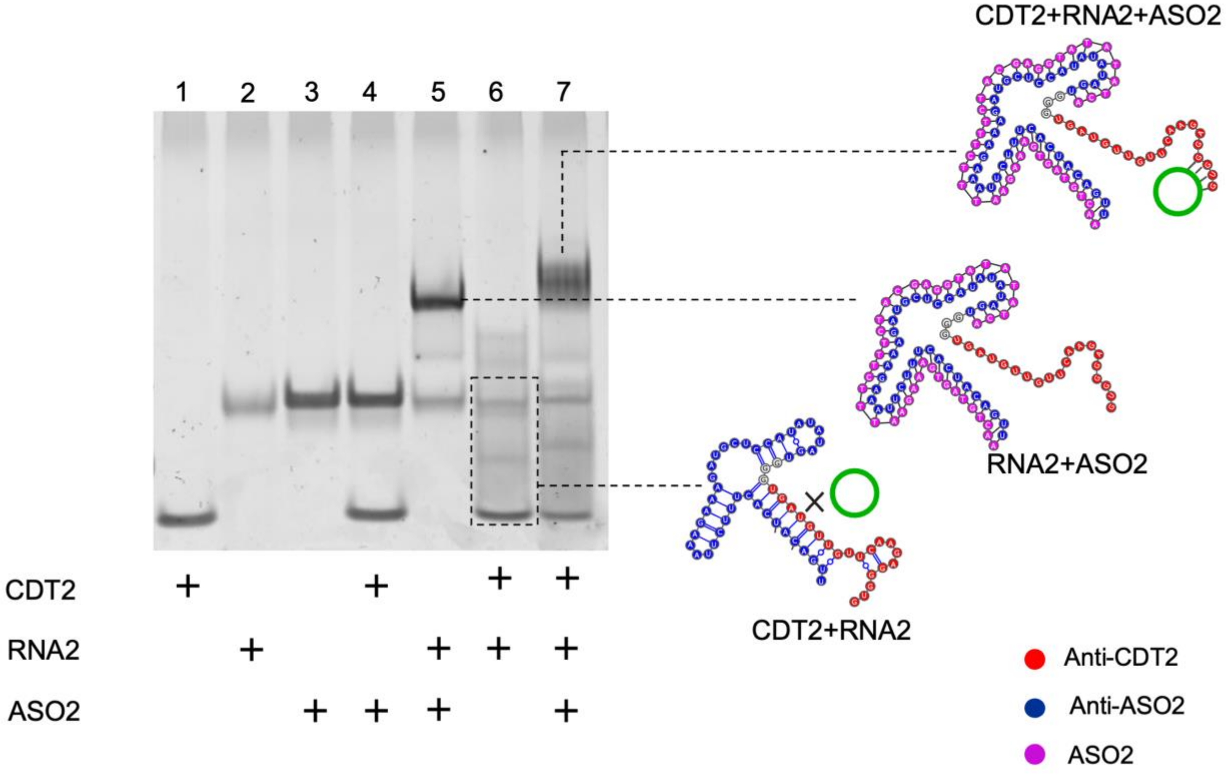

Figure S6. Native gel shift assay demonstrating that ASO2 enhanced hybridization between CDT2 and structured RNA2. In lane 6, CDT2 was incubated with 63 nt RNA2 alone, resulting in minimal complex formation, as indicated by the predominance of unshifted CDT2. In contrast, lane 7 showed that the addition of ASO2 promoted structural reorganization of RNA2, enabling efficient binding to CDT2 and resulting in a prominent shifted band. Corresponding secondary structure models (right) illustrate how ASO2 binding (magenta) exposed the Anti-CDT2 region (red) by disrupting competing intramolecular structures formed by the Anti-ASO2 region (blue), thus facilitating CDT hybridization.

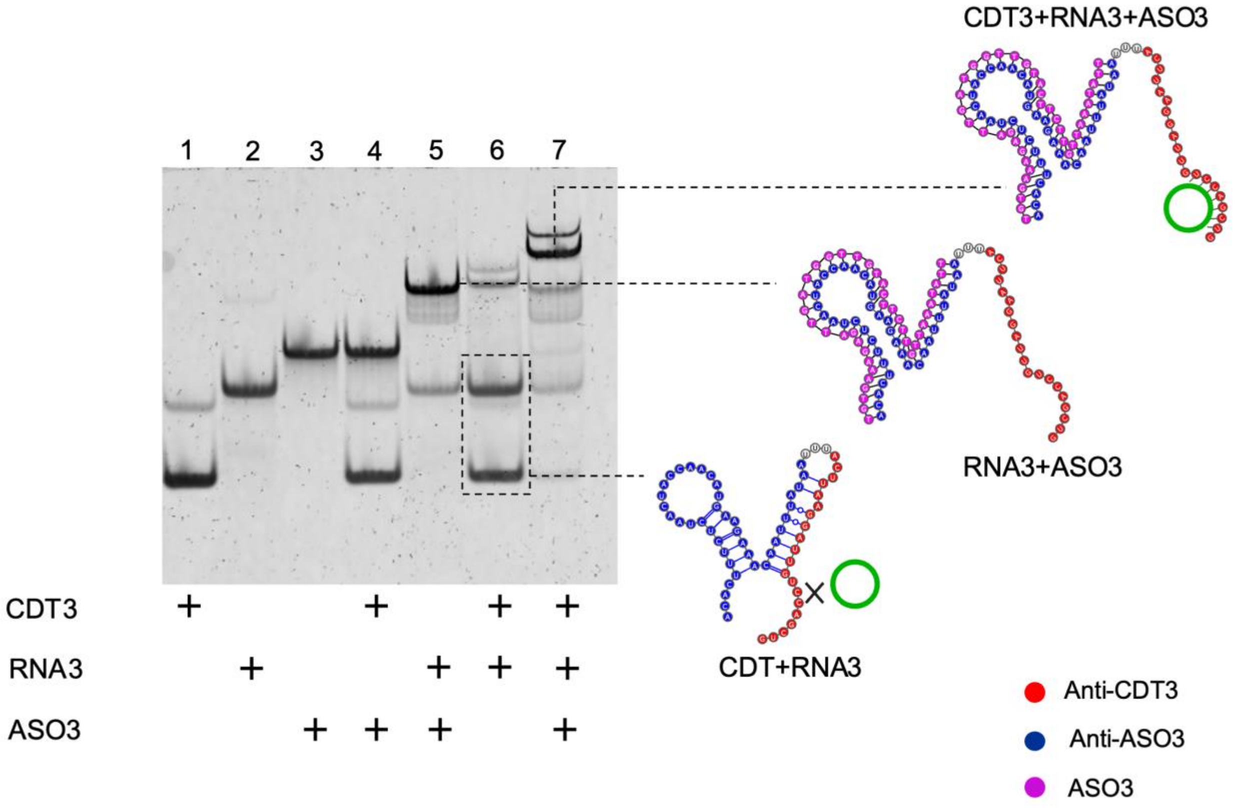

Figure S7. Native gel shift assay demonstrating that ASO3 enhanced hybridization between CDT3 and structured RNA3. In lane 6, CDT3 was incubated with 63 nt RNA3 alone, resulting in minimal complex formation, as indicated by the predominance of unshifted CDT3. In contrast, lane 7 showed that the addition of ASO3 promoted structural reorganization of RNA3, enabling efficient binding to CDT3 and resulting in a prominent shifted band. Corresponding secondary structure models (right) illustrate how ASO3 binding (magenta) exposed the Anti-CDT3 region (red) by disrupting competing intramolecular structures formed by the Anti-ASO3 region (blue), thus facilitating CDT hybridization.

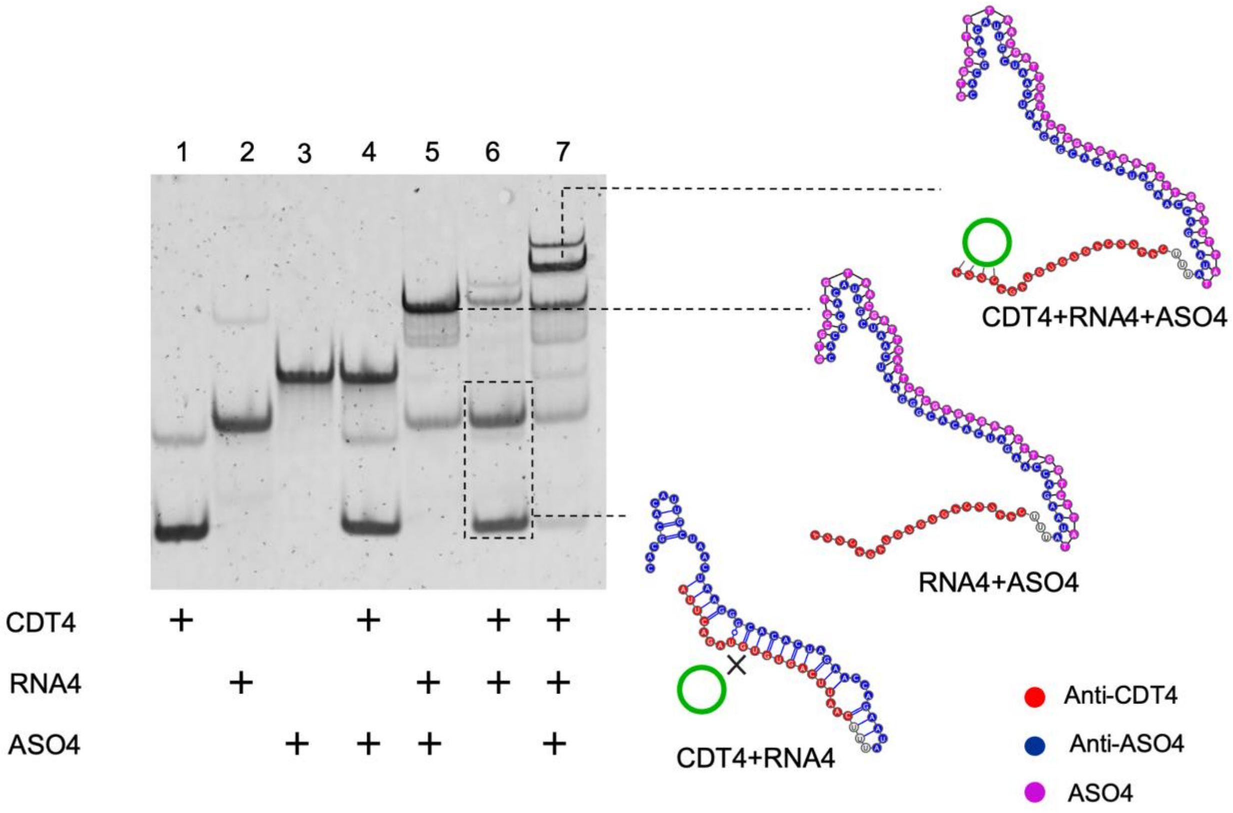

Figure S8. Native gel shift assay demonstrating that ASO4 enhanced hybridization between CDT4 and structured RNA4. In lane 6, CDT4 was incubated with 63 nt RNA4 alone, resulting in minimal complex formation, as indicated by the predominance of unshifted CDT4. In contrast, lane 7 showed that the addition of ASO4 promoted structural reorganization of RNA4, enabling efficient binding to CDT4 and resulting in a prominent shifted band. Corresponding secondary structure models (right) illustrate how ASO4 binding (magenta) exposed the Anti-CDT4 region (red) by disrupting competing intramolecular structures formed by the Anti-ASO4 region (blue), thus facilitating CDT hybridization.

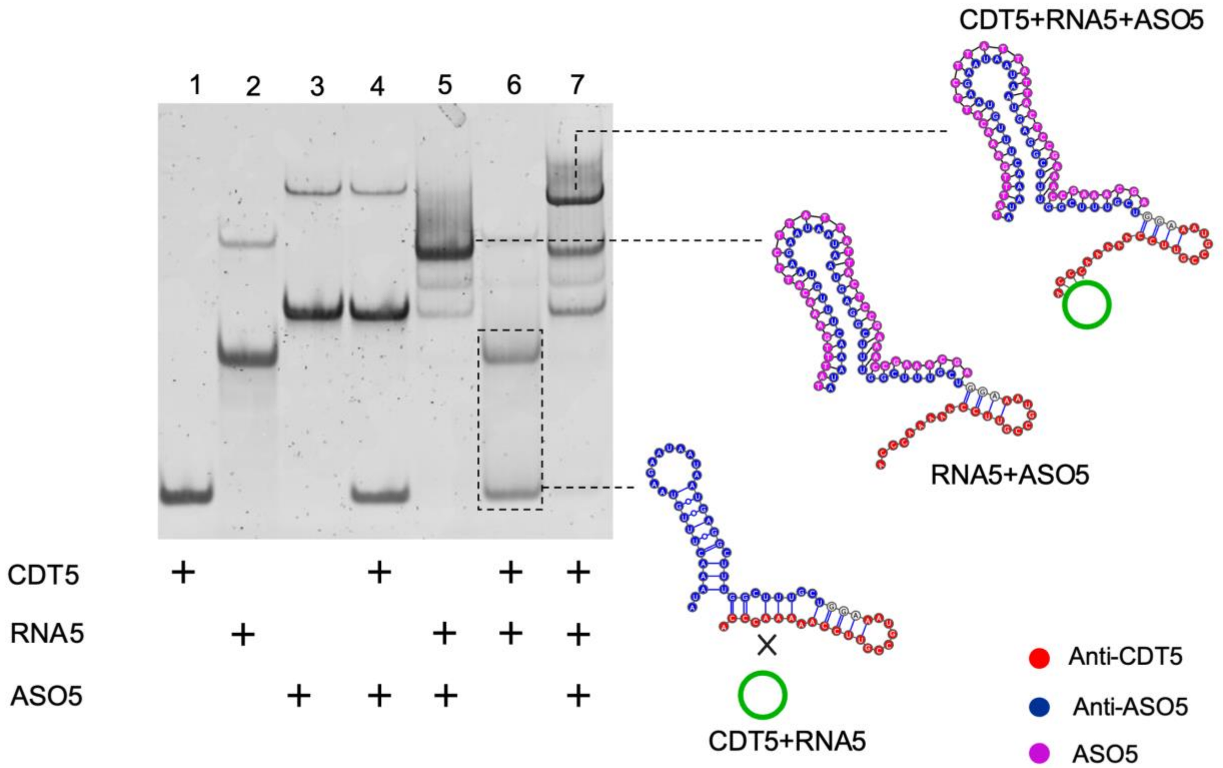

Figure S9. Native gel shift assay demonstrating that ASO5 enhanced hybridization between CDT5 and structured RNA5. In lane 6, CDT5 was incubated with 63 nt RNA5 alone, resulting in minimal complex formation, as indicated by the predominance of unshifted CDT5. In contrast, lane 7 showed that the addition of ASO5 promoted structural reorganization of RNA5, enabling efficient binding to CDT5 and resulting in a prominent shifted band. Corresponding secondary structure models (right) illustrate how ASO5 binding (magenta) exposed the Anti-CDT5 region (red) by disrupting competing intramolecular structures formed by the Anti-ASO5 region (blue), thus facilitating CDT hybridization.

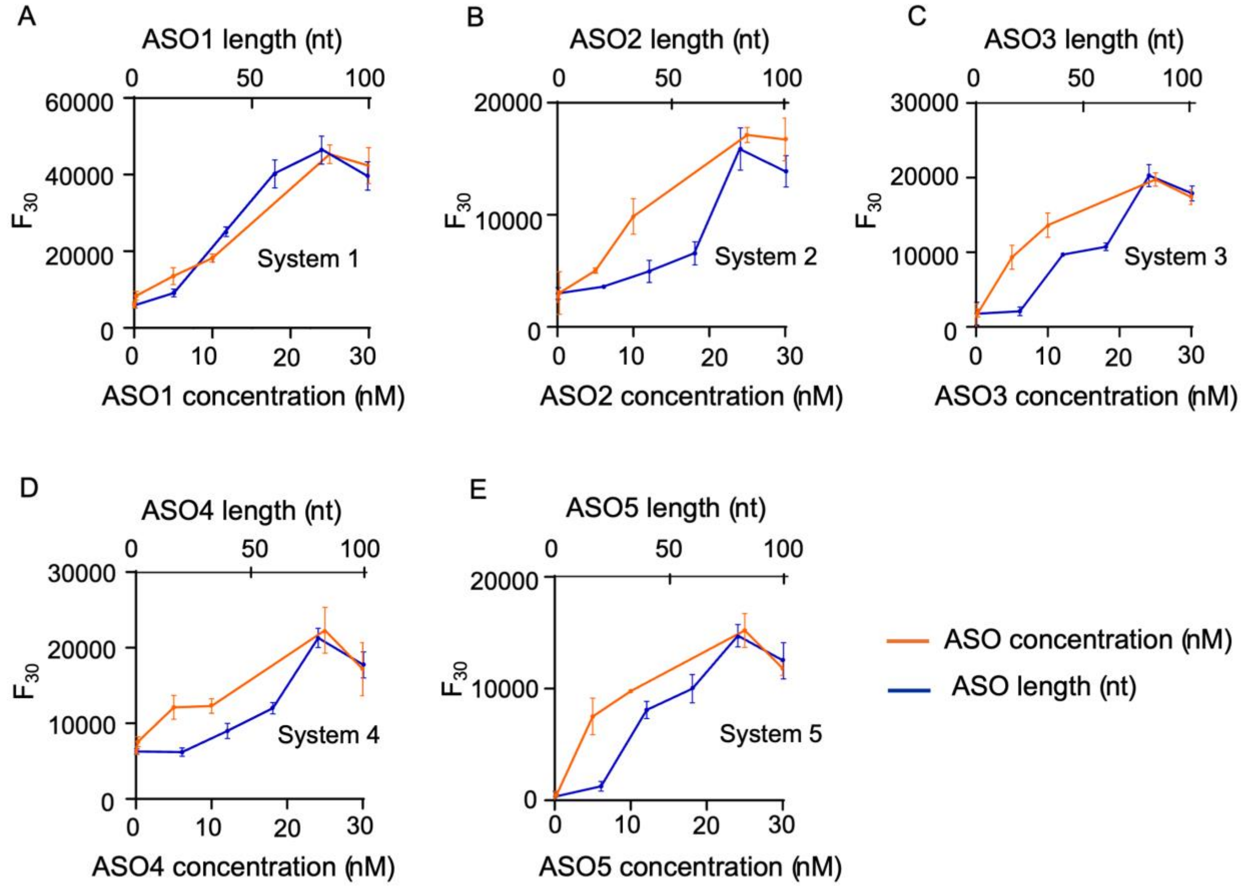

Figure S10. Coupled DNAzyme-RCA reactions with ASO length and concentration titration for all 5 systems. Normalized fluorescence vs ASO length vs ASO concentration showing 80 nt blocker to be the optimal length, and 25 nM or 5X ASO to be the optimal concentration.

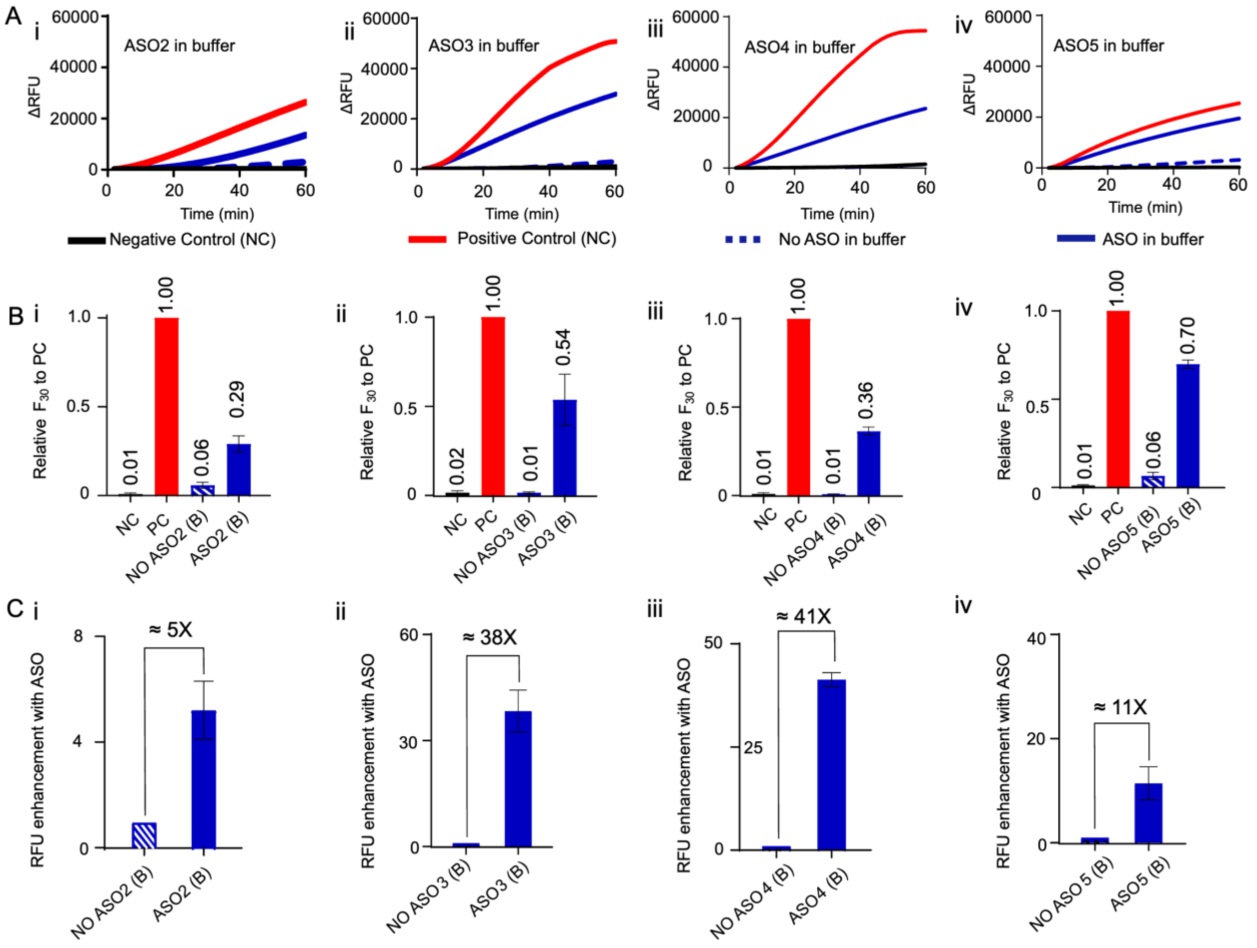

Figure S11. Respective ASOs enhance RCA signal following DNAzyme-mediated cleavage of structured RNA in buffer. (A) (i-iv) Real-time RCA fluorescence curves show that ASOs substantially increases signal output compared to reactions without ASOs for system 2-5. (B) (i-iv) Relative fluorescence units (RFU) at 30 minutes (F_{30}) normalized to the positive control (PC) in buffer. (C) (i-iv) Fold enhancement in RFU by ASOs, calculated by dividing F_{30} with ASOs by F_{30} without ASOs.

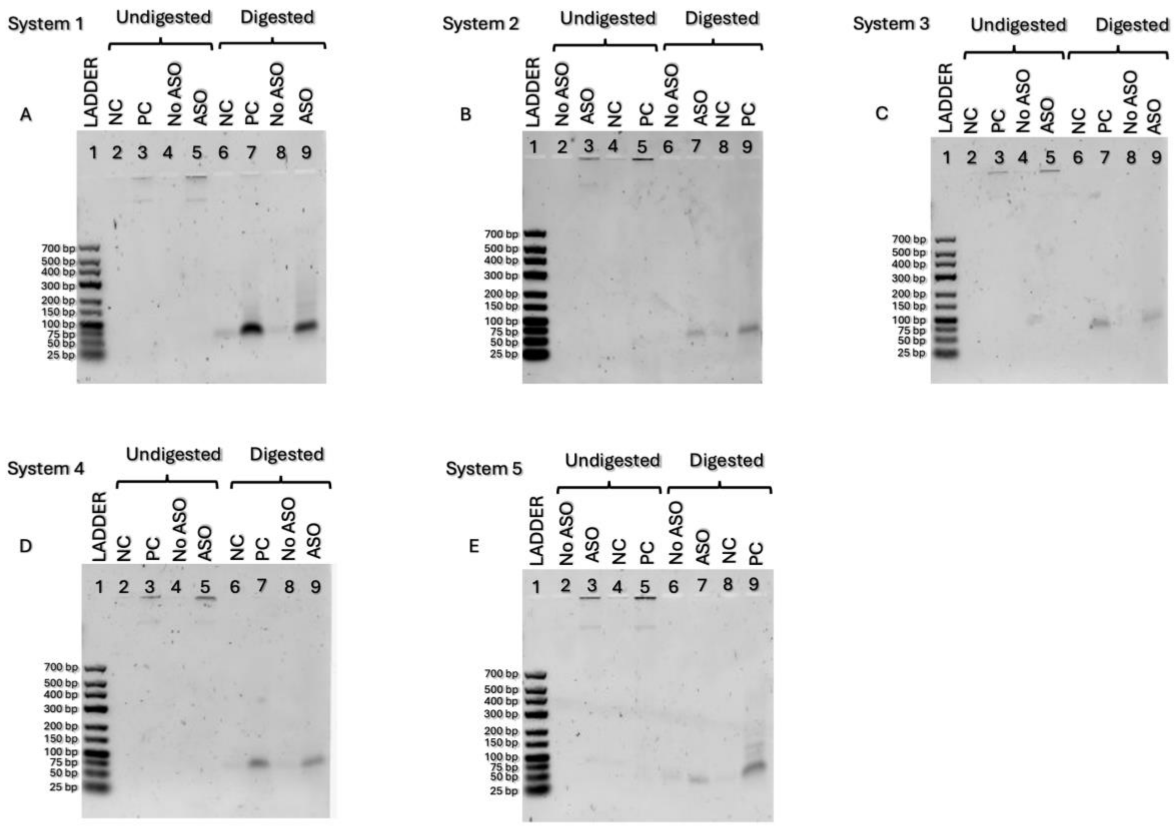

Figure S12. (A-E) Undigested and digested RCA products from four conditions across five systems, resolved on a 1.5% agarose gel. Presence of clearly visible bands in PC and ASO added conditions denote the generation of RCA products. The digestion of RCA products generates 51-nt monomeric products. Undigested RCA products remain in the loading well. Absence of bands for NC and No ASO conditions denote the absence poor generation of RCA products. Lane 1 contains DNA ladders corresponding to 700, 500, 400, 300, 200, 150, 100, 75, 50 and 25 base pairs.

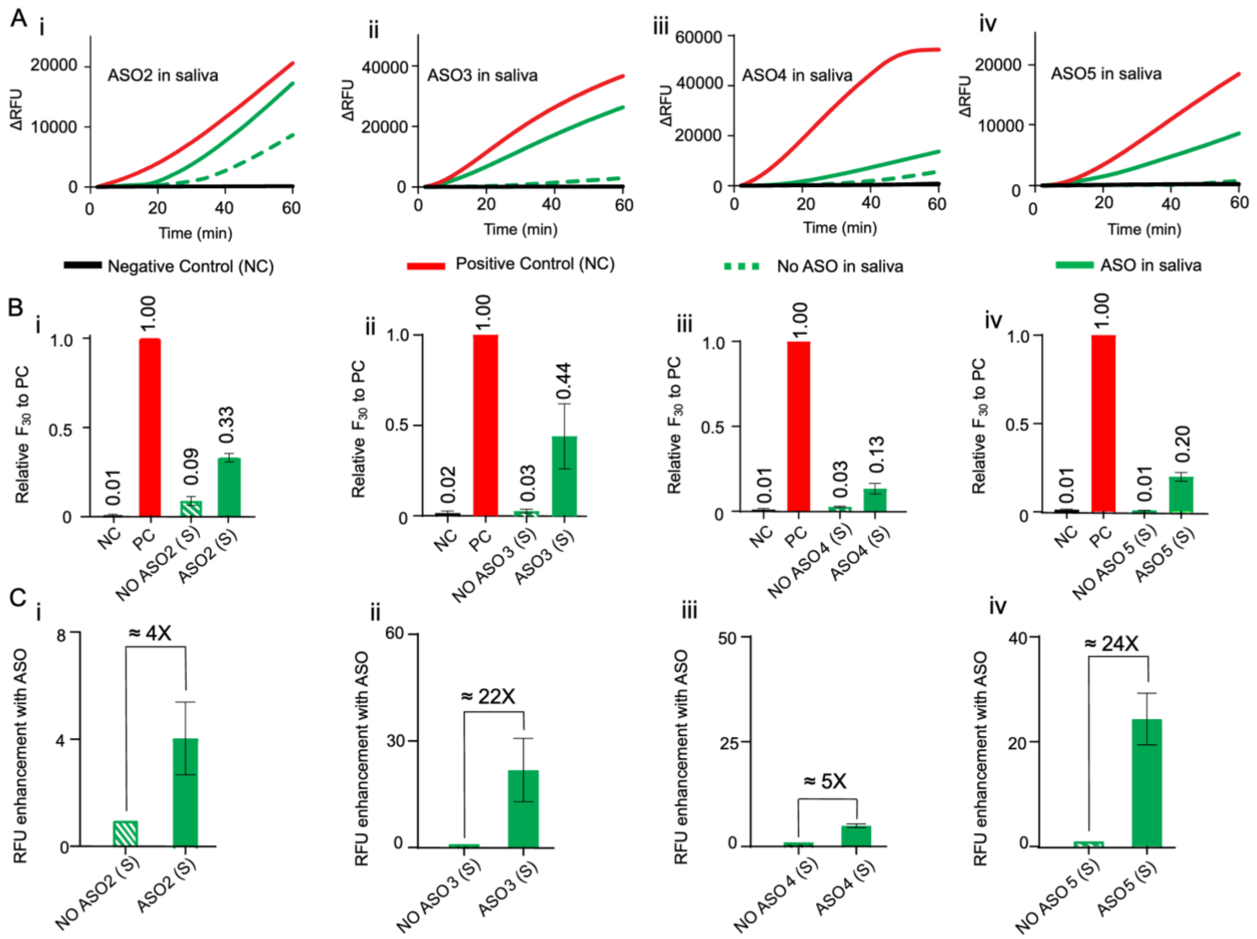

Figure S13. Respective ASOs enhance RCA signal following DNAzyme-mediated cleavage of structured RNA in 50% negative pooled saliva. (A) (i-iv) Real-time RCA fluorescence curves show that ASOs substantially increases signal output compared to reactions without ASOs for system 2-5. (B) (i-iv) Relative fluorescence units (RFU) at 30 minutes (F_{30}) normalized to the positive control (PC). (C) (i-iv) Fold enhancement in RFU by ASOs, calculated by dividing F_{30} with ASOs by F_{30} without ASOs.

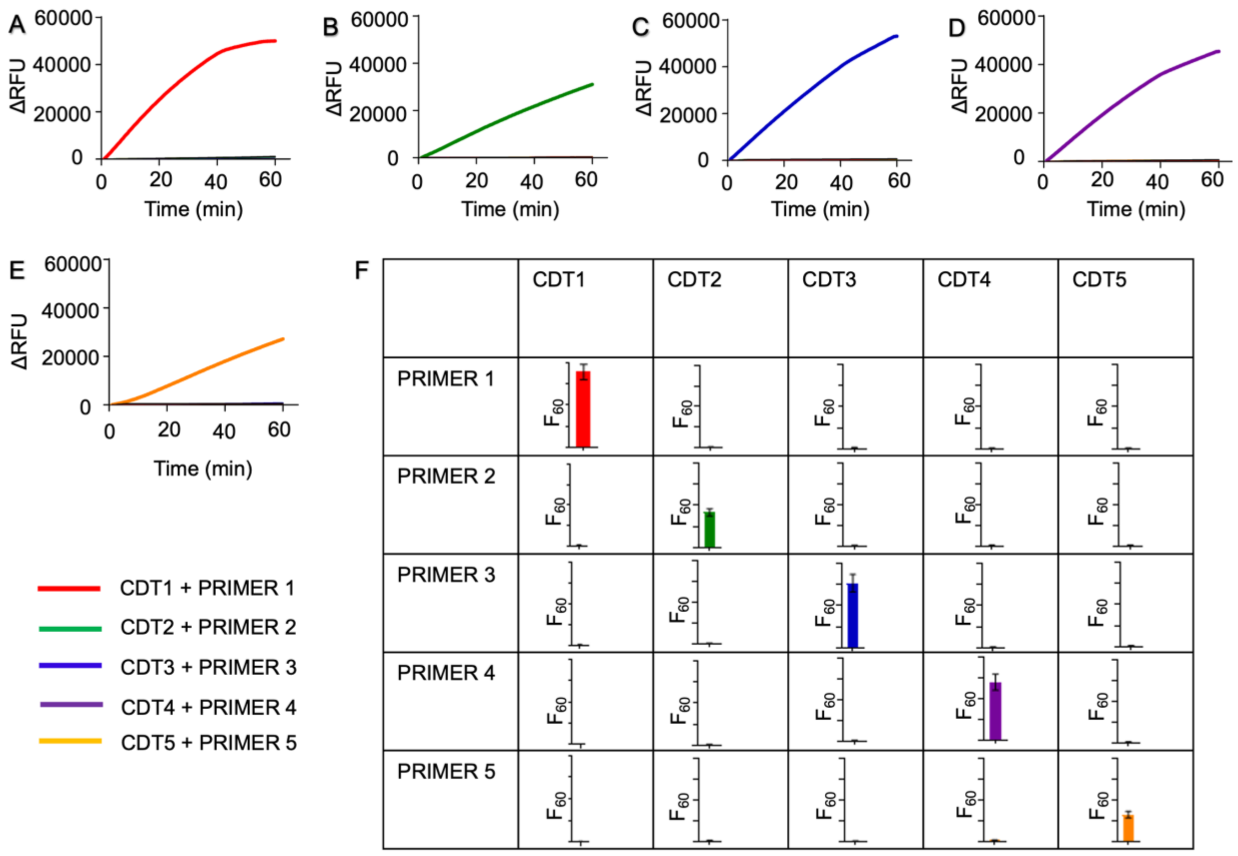

Figure S14. RCA selectivity experiment to show how changes to the primer sequence affects CDT binding. (A-E) CDT1-5 were cross-hybridized with primers 1-5 and the RCA signal was monitored over 60 minutes. Fluorescence is observed only when primers matched their respective CDTs, confirming the high target selectivity of the CDTs. (F) summarizes F_{60} for each RCA reaction.

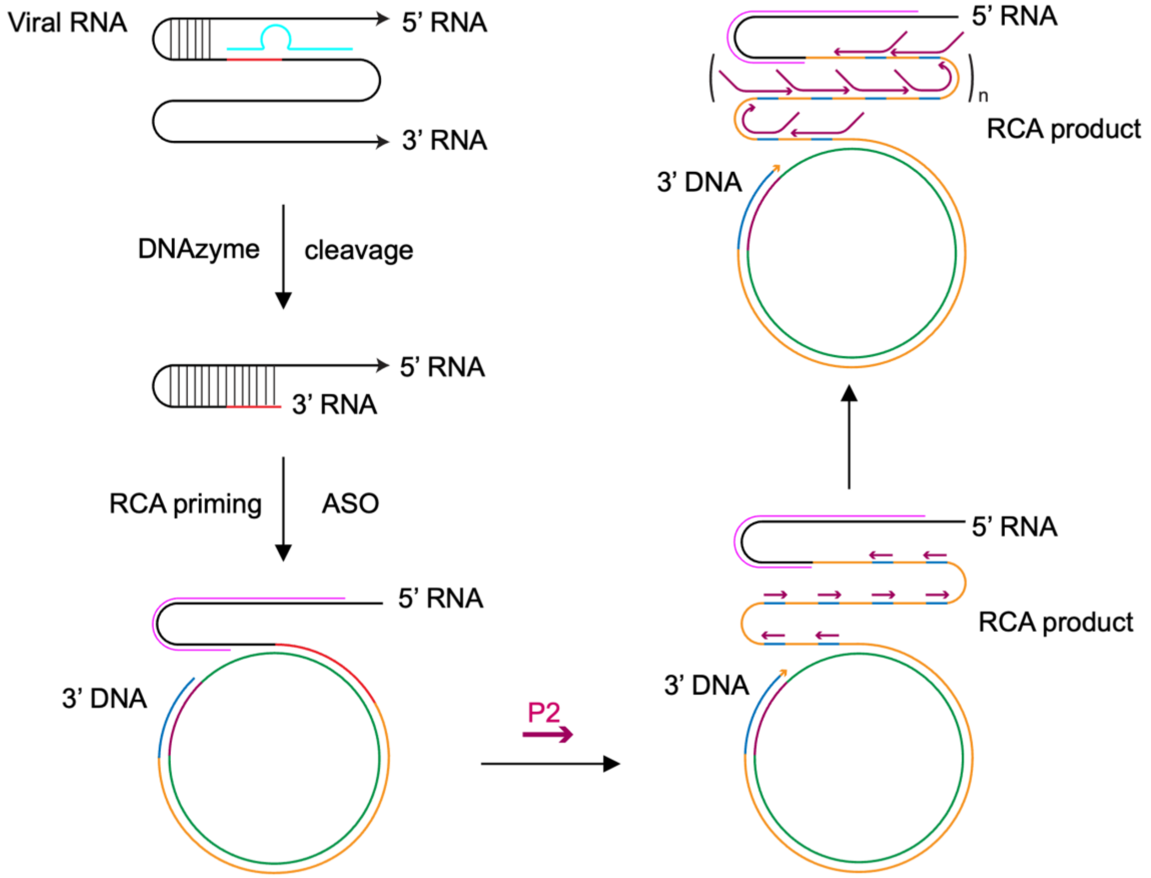

Figure S15. Schematic of the quasi-exponential rolling circle amplification (QE-RCA) reaction used in this study. A 10–23 DNAzyme cleaves structured viral RNA, generating 5′ and 3′ fragments. The 5′ RNA fragment is restructured by an antisense oligonucleotide (ASO, magenta), which exposes the CDT-binding site and enables priming on the circular DNA template (CDT). RCA proceeds through phi29 DNA polymerase, generating a long single-stranded DNA product. A secondary primer (P2, purple) then binds to repeated sequences within the RCA product and initiates further extension, producing branched amplification products. This P2-driven step transforms linear RCA into a quasi-exponential reaction, significantly increasing signal output for enhanced detection sensitivity.

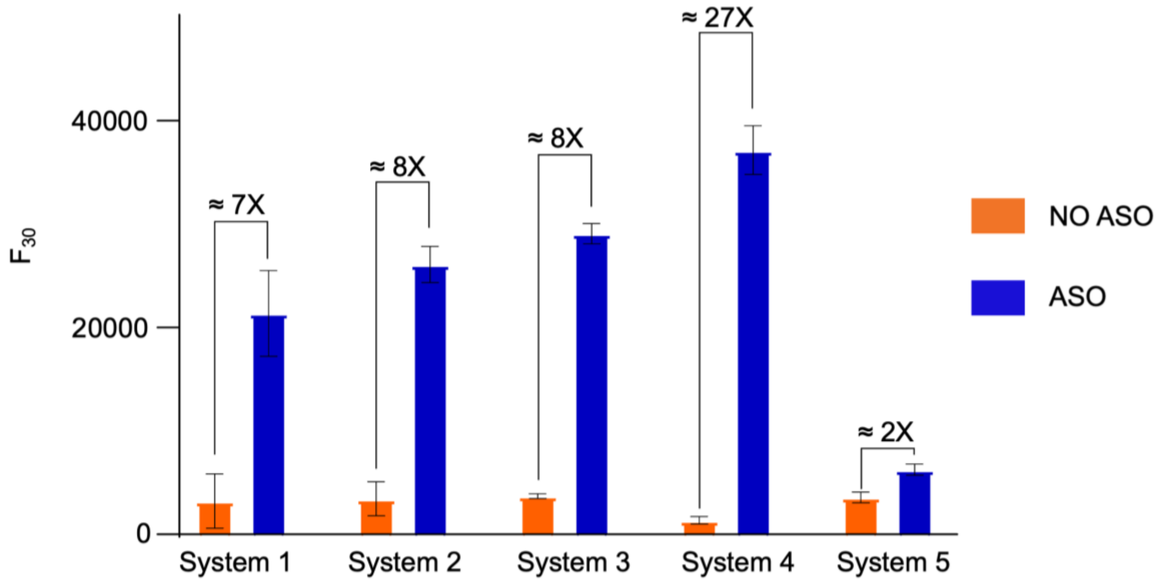

Figure S16. Bar plot showing F_{30} values for all 5 systems with and without respective ASOs using quasi-exponential RCA in 50% negative pooled saliva.

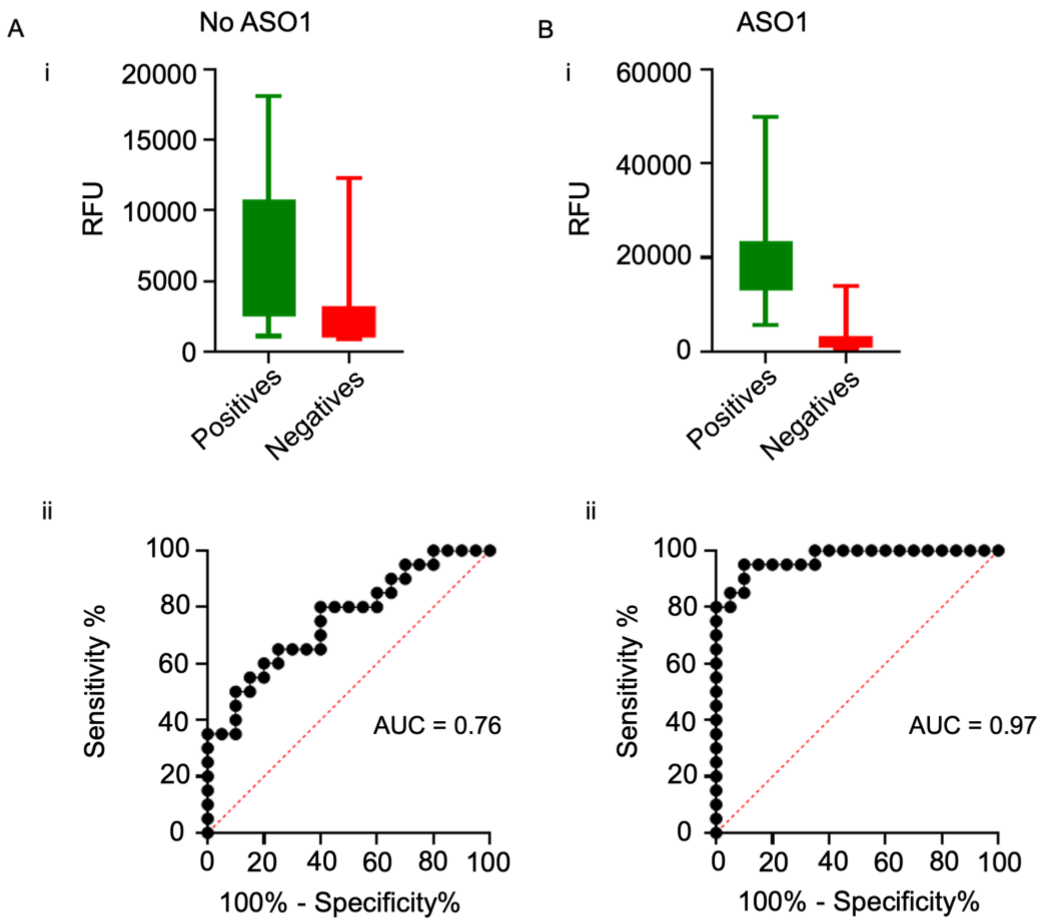

Figure S17. (A) Box plot showing distribution (minimum, maximum, interquartile range and median) of the F_{60} values for 20 positive (green) patient saliva samples and 20 negative (red) patient saliva samples with and without ASO1 conditions. (B) Receiver Operating Characteristic (ROC) plot of coupled assay with and without ASO1 using mean F_{60} and cut-off value from Panel A. (D) RFU average of each sample with and without ASO1 conditions.

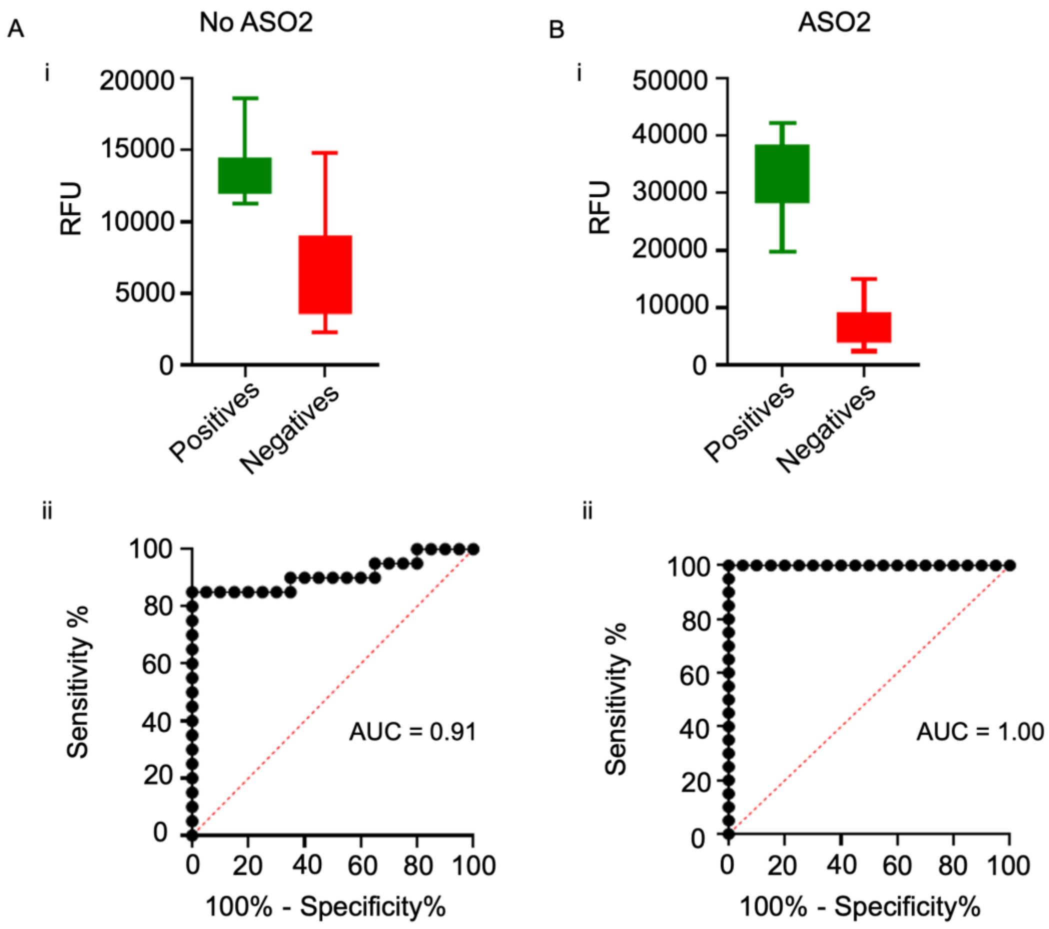

Figure S18. (A) Box plot showing distribution (minimum, maximum, interquartile range and median) of the F_{60} values for 20 positive (green) patient saliva samples and 20 negative (red) patient saliva samples with and without ASO2 conditions. (B) Receiver Operating Characteristic (ROC) plot of coupled assay with and without ASO2 using mean F_{60} and cut-off value from Panel A. (D) RFU average of each sample with and without ASO2 conditions.

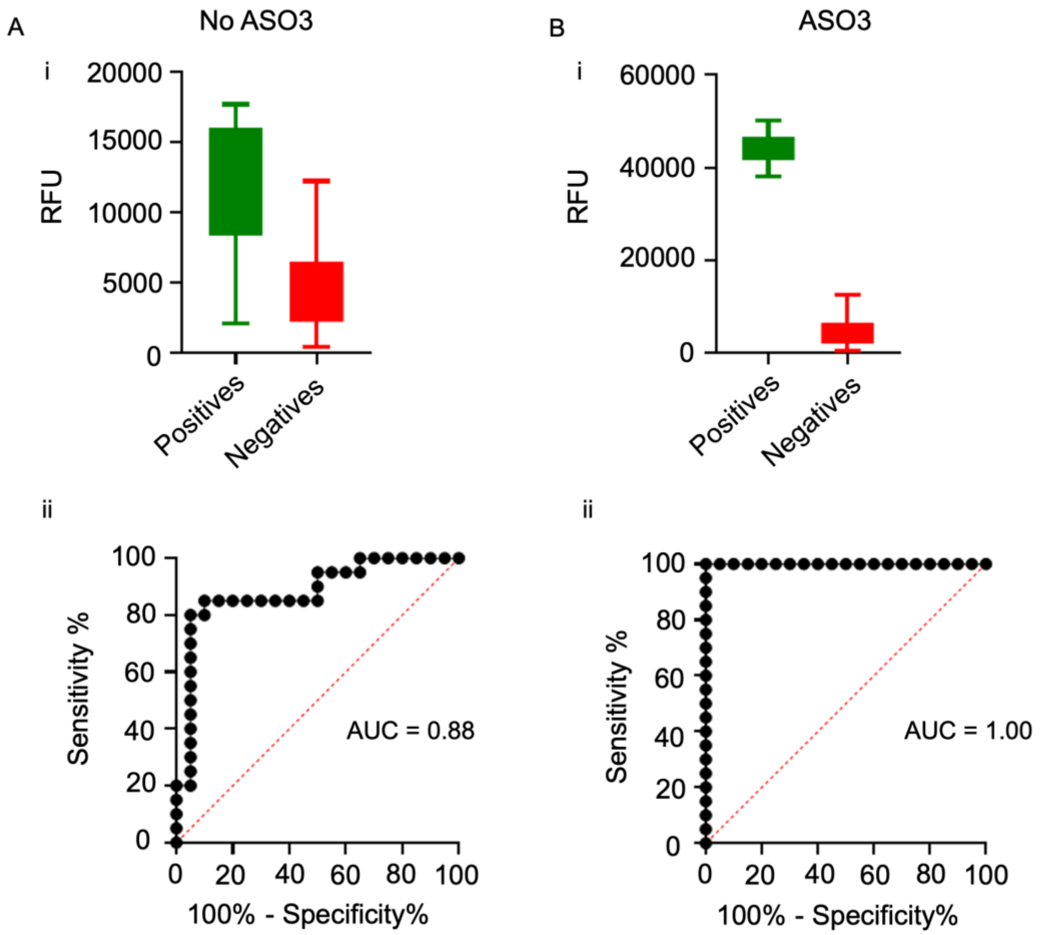

Figure S19. (A) Box plot showing distribution (minimum, maximum, interquartile range and median) of the F_{60} values for 20 positive (green) patient saliva samples and 20 negative (red) patient saliva samples with and without ASO3 conditions. (B) Receiver Operating Characteristic (ROC) plot of coupled assay with and without ASO3 using mean F_{60} and cut-off value from Panel A. (D) RFU average of each sample with and without ASO3 conditions.

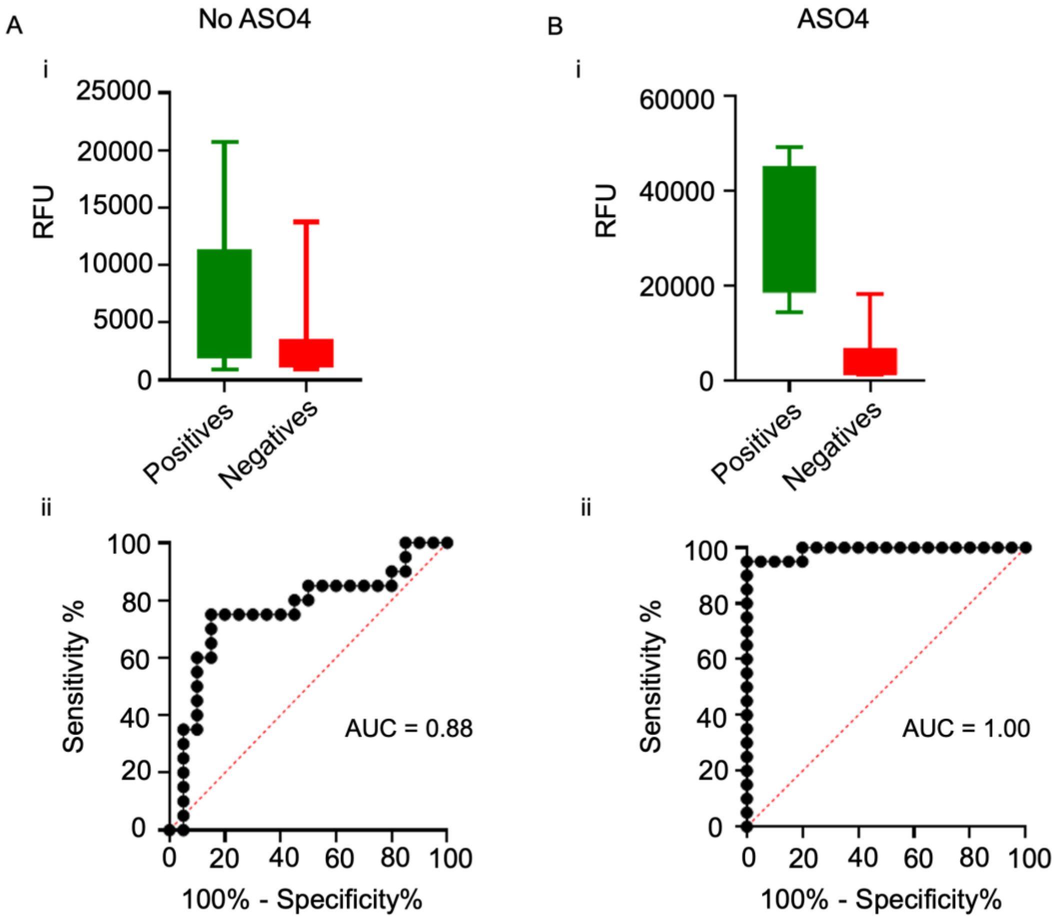

Figure S20. (A) Box plot showing distribution (minimum, maximum, interquartile range and median) of the F_{60} values for 20 positive (green) patient saliva samples and 20 negative (red) patient saliva samples with and without ASO4 conditions. (B) Receiver Operating Characteristic (ROC) plot of coupled assay with and without ASO4 using mean F_{60} and cut-off value from Panel A. (D) RFU average of each sample with and without ASO4 conditions.

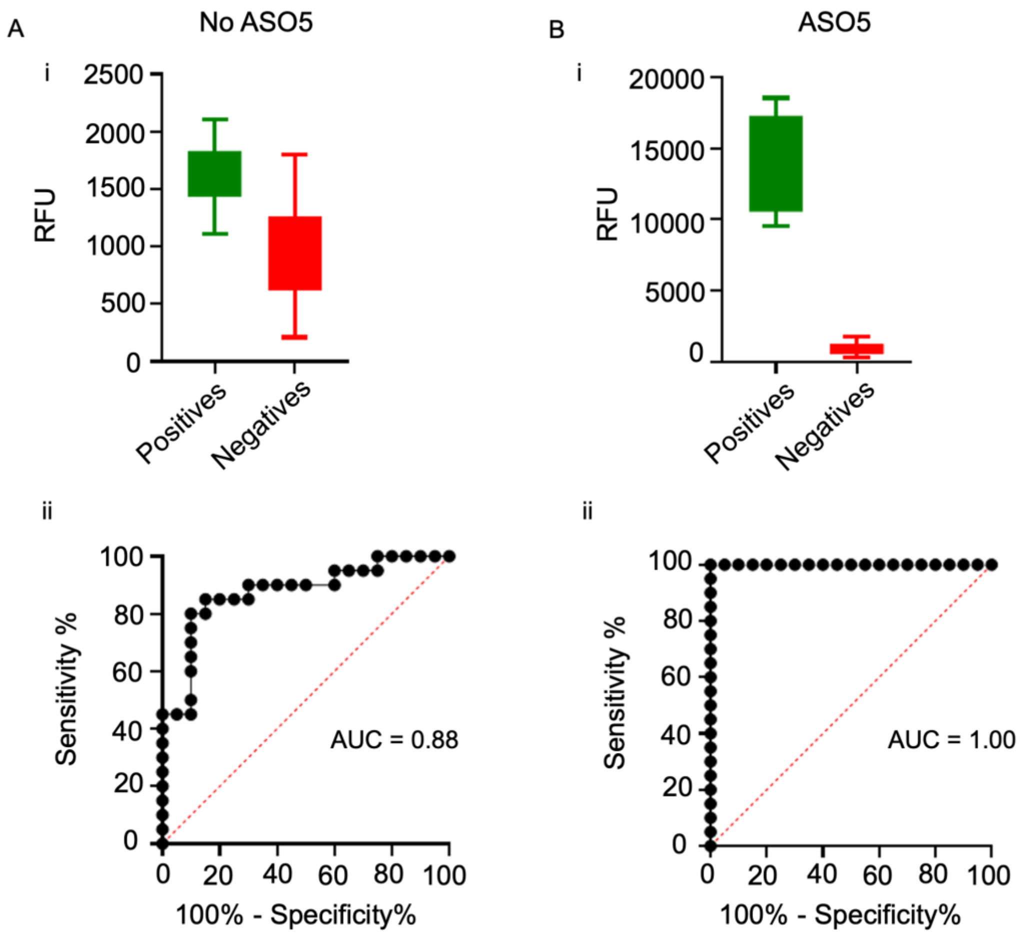

Figure S21. (A) Box plot showing distribution (minimum, maximum, interquartile range and median) of the F_{60} values for 20 positive (green) patient saliva samples and 20 negative (red) patient saliva samples with and without ASO5 conditions. (B) Receiver Operating Characteristic (ROC) plot of coupled assay with and without ASO5 using mean F_{60} and cut-off value from Panel A. (D) RFU average of each sample with and without ASO5 conditions.

Raw gel images

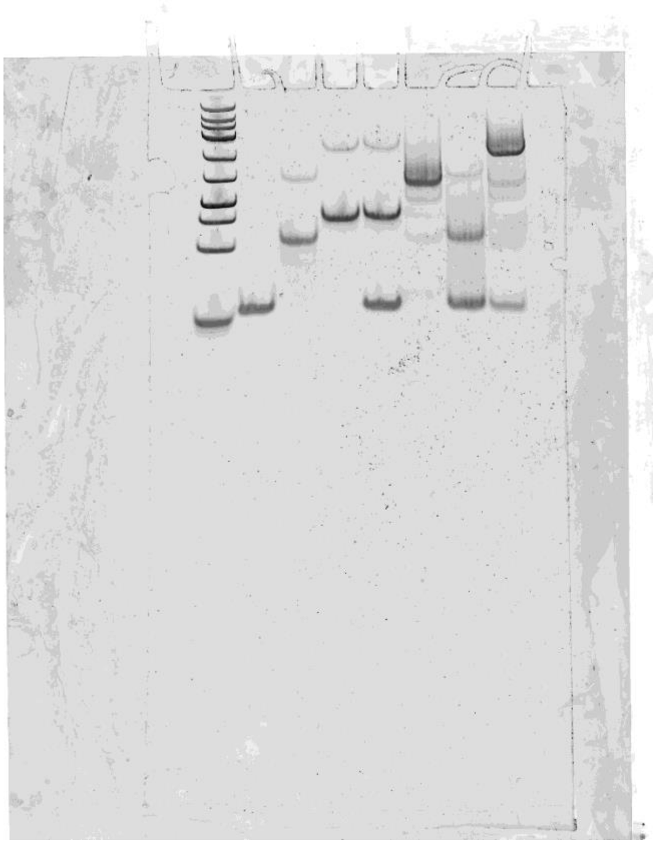

Unprocessed image for gel in Figure 3.

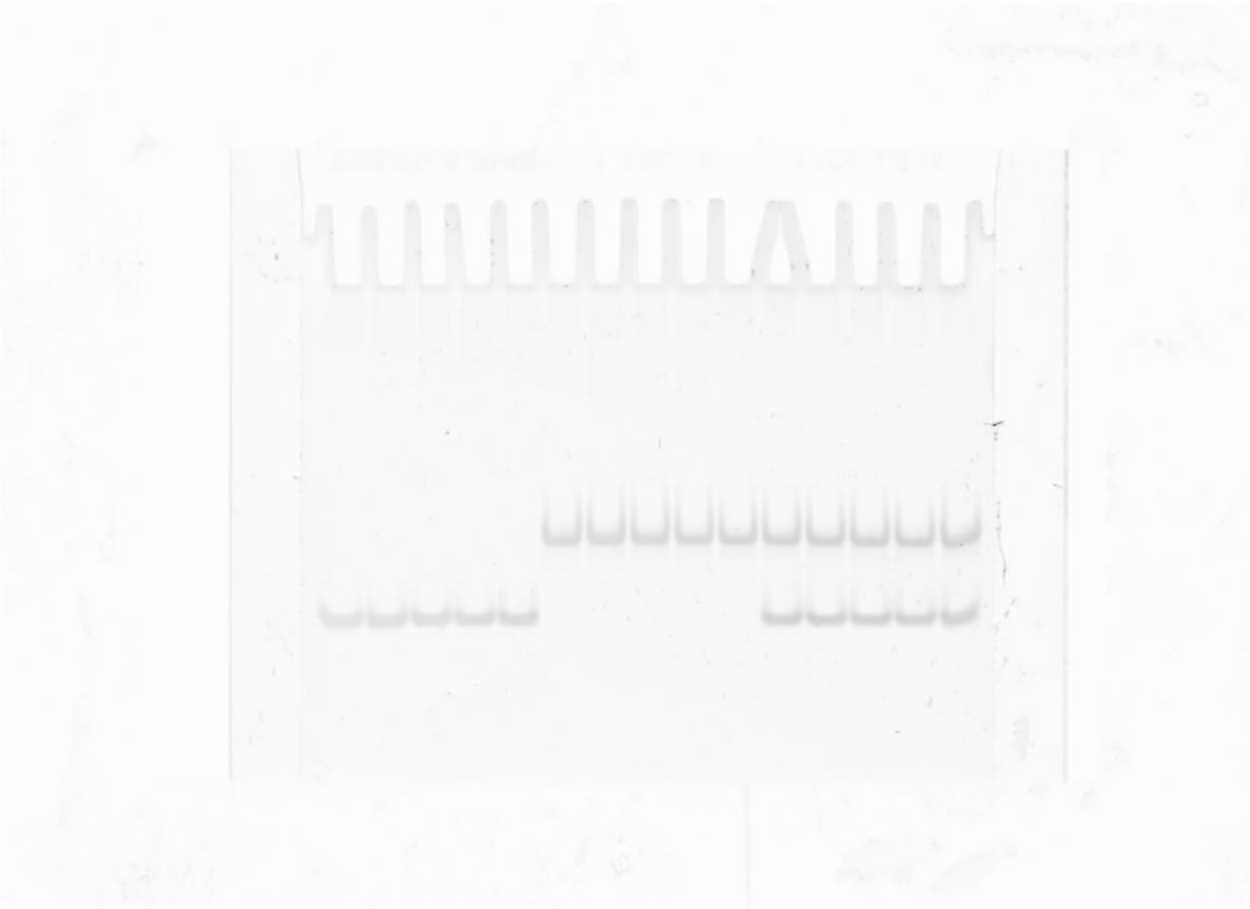

Unprocessed image for gel in Figure S5.

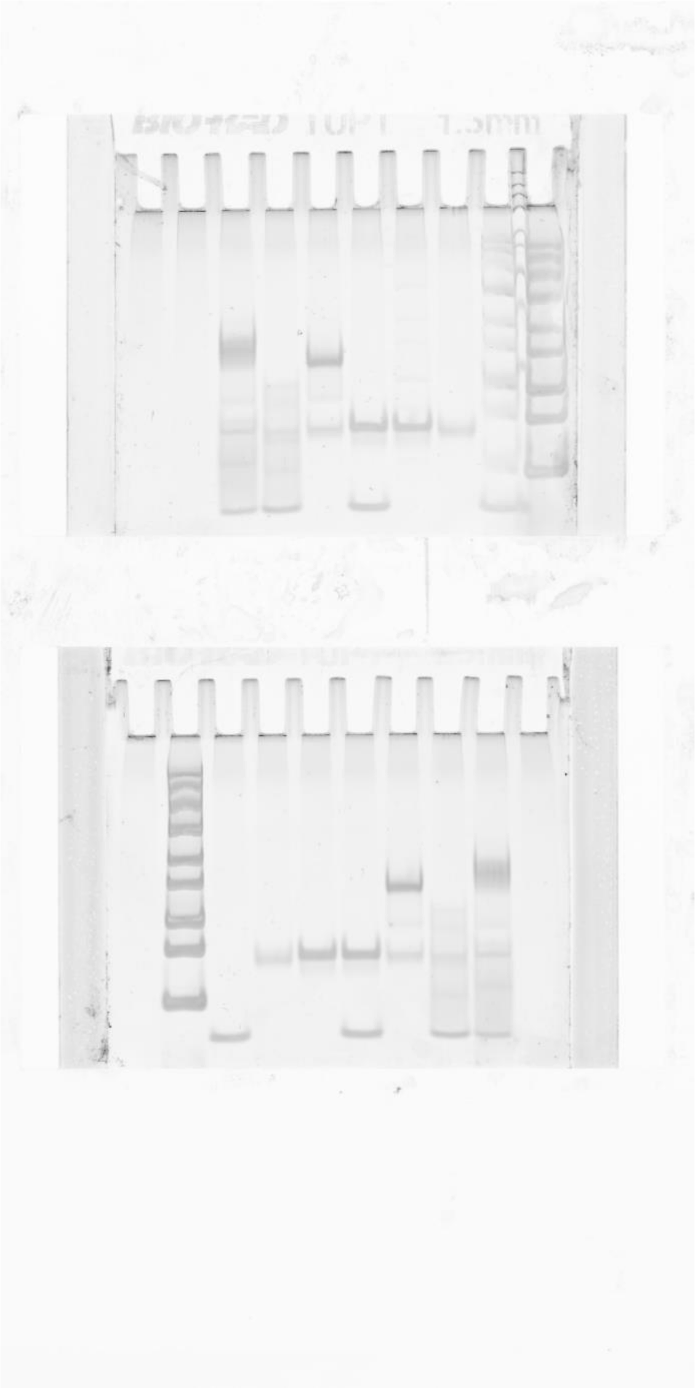

Unprocessed image for gel in Figure S6. Top and bottom gels are repeats of the same gel. Top gel had a spillover of DNA ladder onto adjacent lane.

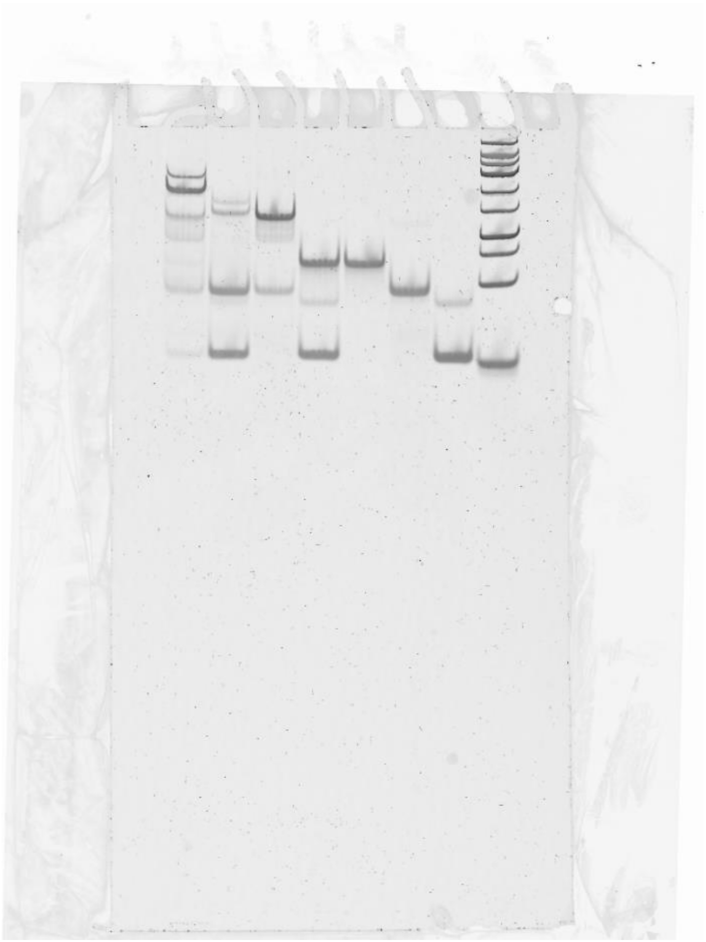

Unprocessed image for gel in Figure S7

.

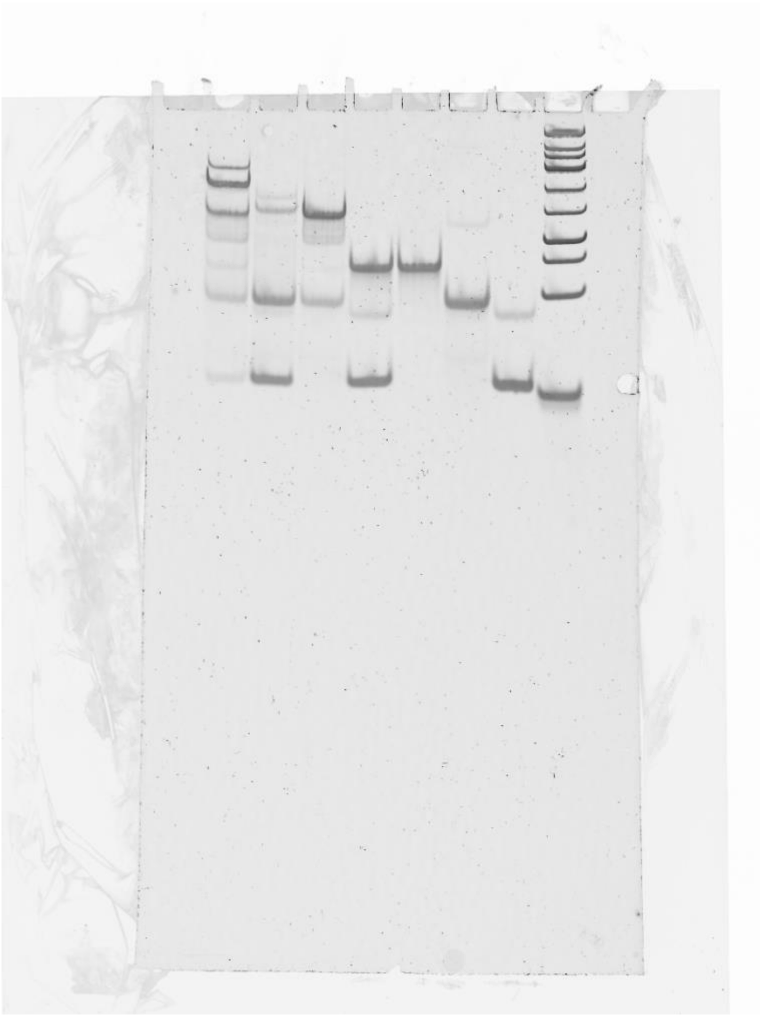

Unprocessed image for gel in Figure S8.

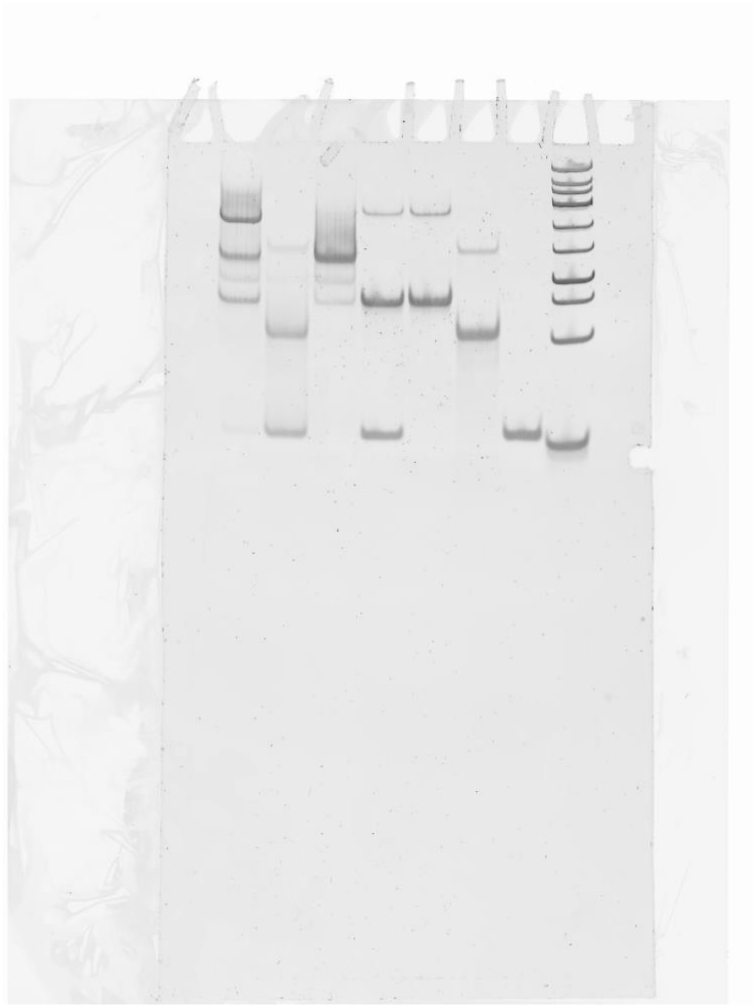

Unprocessed image for gel in Figure S9.

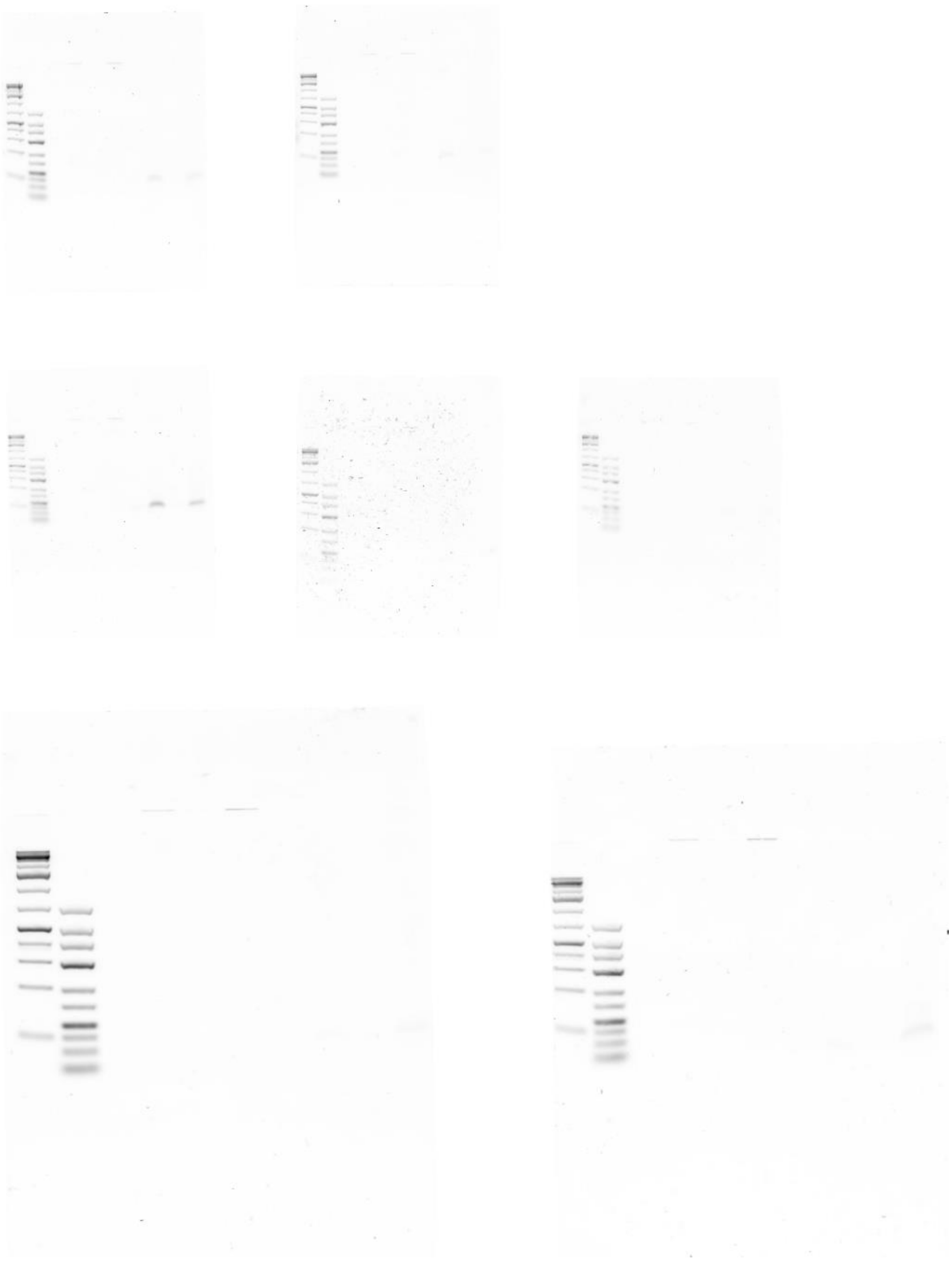

Unprocessed image for gel in Figure S12.

References

[1] D. R. E. Ranoa, R. L. Holland, F. G. Alnaji, K. J. Green, L. Wang, C. B. Brooke, M. D. Burke, T. M. Fan, P. J. Hergenrother, *bioRxiv.* **2020**, 2020.06.18.159434.

[2] D. White, J. Gu, C.-J. Steinberg, D. Yamamura, B. J. Salena, C. Balion, C. D. M. Filipe, A. Capretta, Y. Li, J. D. Brennan, *Sci. Rep.* **2022**, *12*, 2806.
